# Dynamin-2 promotes Atg9A retrieval from phagophores during autophagy

**DOI:** 10.1242/bio.062632

**Published:** 2026-10-16

**Authors:** Andrew Caliri, Alejandro Martorell-Riera, Panagiota Kolitsida, Akash Saha, Cinta Iriondo-Martinez, Samuel Itskanov, Janos Steffen, Carla M. Koehler, Alexander M. van der Bliek

**Affiliations:** ^1^ Department of Biological Chemistry, David Geffen School of Medicine at UCLA, Los Angeles, CA 90095, USA; ^2^ Department of Chemistry and Biochemistry, UCLA, Los Angeles, CA 90095, USA

**Keywords:** Dynamin, Autophagy, Endophilin, Atg9A, LC3

## Abstract

Autophagy involves the rapid growth of phagophores through membrane addition. This growth is triggered by vesicles containing the Atg9A protein. However, Atg9A is not incorporated into mature autophagosomes. We now demonstrate that Dynamin-2 (Dnm2) colocalizes with the BAR domain protein endophilin B1 (EndoB1/Bif-1/SH3GLB1) and other autophagy proteins when autophagy is induced. Our data suggest that Atg9A is retrieved from phagophores via fission, with help from Dnm2. Blocking Atg9A recycling, by mutating Dnm2, using RNA interference, or applying chemical inhibitors, causes Atg9A to remain in autophagosomes and be degraded during autophagy. Overall, these findings provide new insights into the roles of Dnm2 in autophagy.

## Teaser

**Summary:** Autophagy requires Atg9A-containing vesicles to seed phagophores, yet Atg9A is absent from mature autophagosomes. We now show that the retrieval of Atg9A from phagophores is mediated by Dnm2.

## INTRODUCTION

Autophagy is a universal process for removing damaged components in the cell and recycling proteins during starvation (Choi et al., 2013). Much has been learned about this process through studying various protein complexes that quickly and intricately assemble autophagosomes, which then fuse with lysosomes for degradation (Hurley and Young, 2017; Levine and Klionsky, 2017; Yamamoto et al., 2023). Autophagosomes are double-membrane compartments that capture cargo destined for lysosomal breakdown (Bento et al., 2016). In the early stages of autophagy, precursors called phagophores form near the endoplasmic reticulum (ER) (Karanasios et al., 2016; Orsi et al., 2012). These phagophores rapidly grow to enclose the material to be degraded in autolysosomes (Chang et al., 2021; Noda, 2021).

Autophagy is triggered either by starvation or by ubiquitinated cargo, such as defective mitochondria and other organelles marked for degradation in autolysosomes. Autophagy receptors such as p62 and NDP52 link ubiquitinated cargo to FIP200 and ULK1 (Ravenhill et al., 2019; Turco et al., 2019), initiating the process that leads to autophagosome formation. Autophagosome membranes originate from Atg9A-containing vesicles derived from the Golgi apparatus, recycling endosomes, and other organelles (Sawa-Makarska et al., 2020). These Atg9A vesicles interact with FIP200 and ULK1 during early stages of autophagy (Stanley et al., 2014). They serve as seeds for autophagosome growth (Olivas et al., 2023). The vesicles expand into a larger envelope around the cargo by adding membranes (Gomez-Sanchez et al., 2018). Atg2 acts as a conveyor, extracting lipids from the ER via interactions with VMP1 and TMEM41B (Yamamoto et al., 2023) and then transferring these lipids to the outer leaflet of the phagophore membrane (Maeda et al., 2019; Osawa et al., 2019; Valverde et al., 2019). To balance the growth of the outer and inner leaflets, excess lipids on the outer surface are moved to the inner surface by Atg9A, which functions as a scramblase (Guardia et al., 2020; Maeda et al., 2020; Matoba et al., 2020). Atg9A is also the only known autophagy protein with transmembrane segments. Interestingly, this protein was found not to be incorporated into mature autophagosomes (Orsi et al., 2012). Instead, Atg9A is likely retrieved from growing phagophores, but the mechanism remains unknown.

In this study, we examined the potential roles of Dynamin-2 (Dnm2) in autophagy. Dnm2 is one of three classic dynamins in mammalian cells (Antonny et al., 2016; Ferguson and De Camilli, 2012; Schmid and Frolov, 2011). It is expressed throughout the body and facilitates membrane scission in various endocytic processes, including the formation of both clathrin- and caveolin-coated vesicles (Conner and Schmid, 2003). Dynamin-1 and dynamin-3 are mainly found in neurons, where they function at presynaptic and postsynaptic densities during endocytosis (Ferguson and De Camilli, 2012). Over time, several additional roles for Dnm2 have been suggested. These include a role in creating exocytic vesicles that bud from the trans Golgi network (TGN) (Jones et al., 1998; Liu et al., 2008), forming actin comets on endocytic vesicles (Lee and De Camilli, 2002; Orth et al., 2002), aiding late-stage cytokinesis (Thompson et al., 2002), supporting late-stage mitochondrial fission (Lee et al., 2016), regenerating autolysosomes during lipid droplet breakdown in hepatocytes (Schulze et al., 2013) and forming ATG9A-containing vesicles from recycling endosomes during autophagy (Soreng et al., 2018; Takahashi et al., 2016). Many of these proposed functions still require further validation, but Dnm2's role in endocytosis has been observed across diverse contexts.

All three dynamins have very similar sequences. Each contains five distinct protein domains: an N-terminal GTPase domain, a middle domain, a pleckstrin homology (PH) domain, a GTPase effector domain (GED), and a proline-rich domain (PRD) (Ferguson and De Camilli, 2012). The middle domain and GED fold back on each other to form a stalk, while the PH domain interacts with membranes, and the PRD binds to SH3 domains in various adaptor proteins (Chappie et al., 2010; Faelber et al., 2011; Ford et al., 2011; Gao et al., 2010a). A large pool of dynamin proteins exists as homomeric tetramers in the cytosol, but a small fraction gathers at specific spots on the plasma membrane to promote membrane scission (Schmid and Frolov, 2011). Multiple contacts between adjacent dynamin molecules at those sites enable assembly into chains, which then form multimeric spirals that wrap around the necks of budding endocytic vesicles and constrict to sever the membrane while hydrolyzing GTP (Schmid and Frolov, 2011).

One key factor in guiding dynamins to different membranes is whether specific binding partners are present. Dynamins interact with PI(4,5)P_2_ at the plasma membrane (Achiriloaie et al., 1999) and with SH3 domain-containing proteins like amphiphysin and endophilin A during endocytosis (Simpson et al., 1999). Cooperative binding between dynamin and amphiphysin or endophilin A has been shown to help tubulate membranes *in vitro* and facilitate membrane severing *in vivo* (Farsad et al., 2001; Meinecke et al., 2013; Renard et al., 2015). Both amphiphysin and endophilin A have isoforms, such as amphiphysin-2 (Bin-1) and endophilin B1 (EndoB1, SH3GLB1 or Bif-1), which might act on other membranes (Farsad et al., 2001; Lee et al., 2002). EndoB1 has been linked to autophagy, either as part of an autophagy protein complex (Takahashi et al., 2007) or by affecting Atg9A vesicle formation at recycling endosomes (Takahashi et al., 2016). While exploring EndoB1's role in autophagy, we observed colocalization of EndoB1 and Dnm2, prompting us to further investigate Dnm2's potential role in autophagy. In this study, we show that Dnm2 facilitates the retrieval of Atg9A from phagophores.

## RESULTS

### Dnm2 and EndoB1 colocalize during autophagy

To test for Dnm2-EndoB1 colocalization, we performed immunofluorescence experiments on wild-type (WT) mouse embryonic fibroblast (MEF) cells treated with or without rapamycin to induce autophagy. These cells were permeabilized with digitonin to enhance the signal from membrane-bound proteins by removing soluble cytosolic proteins (Liu et al., 1998). We observed a significant increase in Dnm2 and EndoB1 colocalization after rapamycin treatment (Fig. 1A), as shown by Manders' correlation coefficient (Fig. 1B) and scatterplots (Fig. S1A,B). To further evaluate these interactions, we performed live-cell imaging of transfected proteins with fluorescent tags, as demonstrated with images (Fig. 1C), Manders' coefficient (Fig. 1D), and scatterplots (Fig. S1C,D). In each case, rapamycin and carbonyl cyanide *m*-chlorophenyl hydrazone (CCCP) significantly increased colocalization between Dnm2 and EndoB1.

**Fig. 1.**
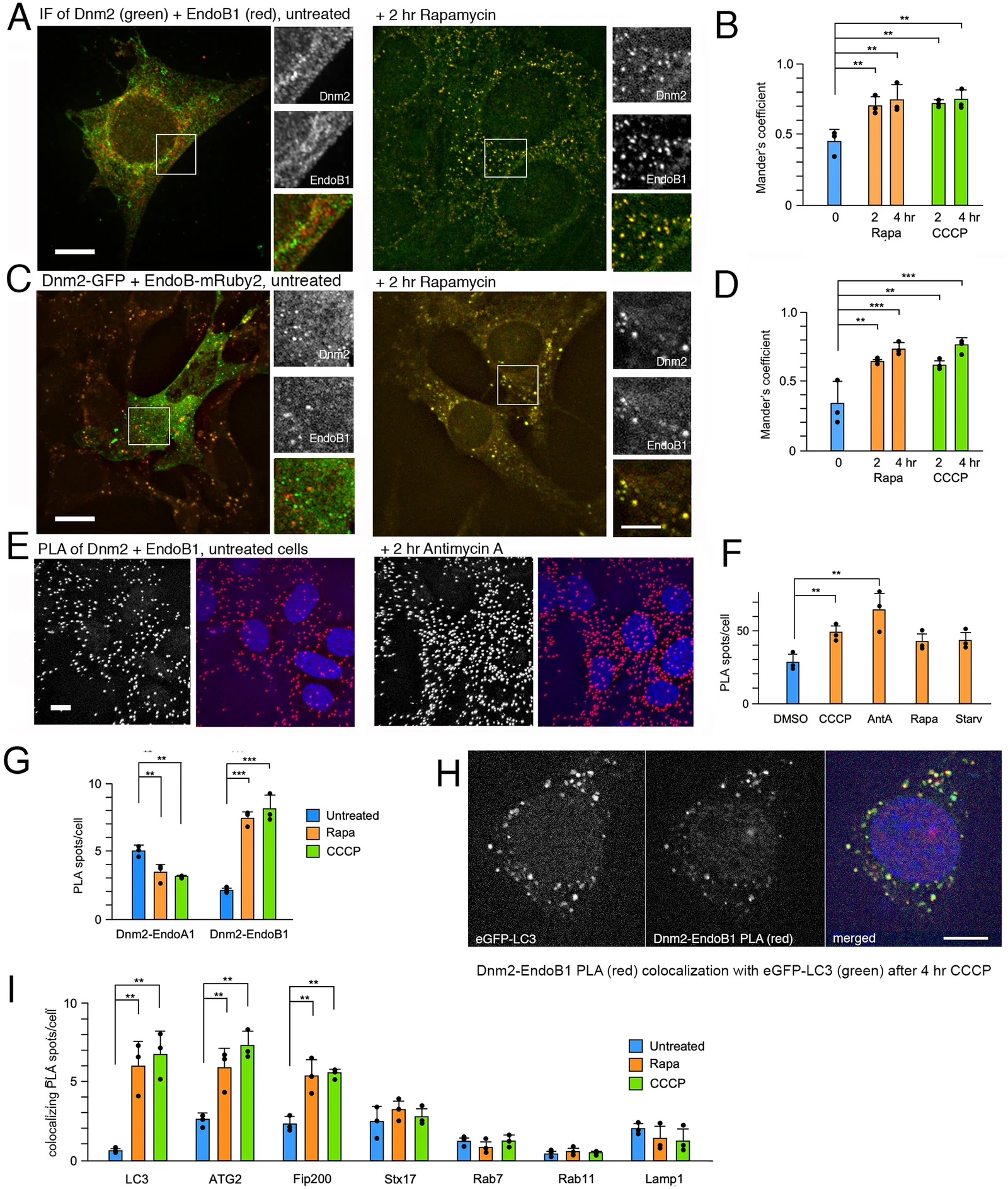
**Colocalization of Dnm2 and EndoB1 with autophagy proteins.** (A) Immunofluorescence of endogenous Dnm2 (green) and EndoB1 (red) in MEFs is shown without or with 2-h rapamycin treatment. To reduce the diffuse cytosolic staining of Dnm2, these cells were incubated with 0.05% digitonin before fixation with paraformaldehyde. Scale bars: 10 μm for whole cells and 5 μm for enlarged portions. (B) Manders' coefficients for colocalization are shown for untreated cells and after 2- or 4-h treatment with rapamycin or CCCP. Average and s.d. are shown for three biological replicates, each determined with five technical repeats. Statistical analysis used one-way analysis of variance (ANOVA) with Dunnett's post hoc test across biological replicates. (C,D) Similar experiments with transfected cells expressing Dnm2-GFP and EndoB1-mRuby2. Scale bars: 10 μm for whole cells and 5 μm for enlarged portions. (E) Colocalization of Dnm2 and EndoB1 tested with PLA shown with or without 2-h antimycin A treatment. Scale bar: 10 μm. (F) Average numbers of PLA spots per cell are shown for Dnm2 and EndoB1 colocalization in untreated MEFs, MEFs treated with CCCP, antimycin A, and rapamycin, and MEFs starved to induce autophagy. The number of spots was counted in 50 cells per experiment. Average and s.d. are shown for three biological replicates, each determined with ten technical repeats. Statistical analysis used one-way ANOVA with Dunnett's post hoc test across biological replicates. (G) The number of Dnm2-EndoB1 PLA spots per cell increases, while Dnm2-EndoA1 PLA spots marginally decrease when autophagy is induced. Average and s.d. are shown for three biological replicates, each determined with 25 technical repeats. Statistical analysis used one-way ANOVA with Dunnett's post hoc test across biological replicates for each PLA protein pair. (H) Images of PLA spots for endogenous Dnm2 and EndoB1 (red) and colocalization with EGFP-LC3 (green) when mitophagy is induced (4 h of CCCP treatment). Scale bar: 10 μm. (I) A larger fraction of Dnm2-EndoB1 PLA spots colocalizes with GFP-tagged LC3, Atg2, and Fip200 when autophagy is induced by rapamycin or CCCP treatment, suggesting that Dnm2 is linked to autophagy. The fraction colocalizing with GFP-tagged LAMP1 decreases and remains low for Stx17, Rab11, and Rab7. Average and s.d. are shown for three biological replicates, each determined with 13-25 technical repeats. Statistical analysis used one-way ANOVA with Dunnett's post hoc test across biological replicates for each set of three conditions per colocalizing protein (**P<0.01; ***P<0.001).

We further examined the colocalization of endogenous Dnm2 and EndoB1 proteins using a proximity ligation assay (PLA). First, we confirmed the specificity of PLA with MEF cells harboring homozygous deletions of the Dnm2 and EndoB1 genes, as well as MEF cells lacking both genes. Western blots verified the absence of Dnm2 and EndoB1 expression in these cell lines (Fig. S1E). The PLA for Dnm2 and EndoB1 in these knockout (KO) cells did not produce any spots (Fig. S1F), indicating that the PLA with Dnm2 and EndoB1 antibodies is highly specific. Additionally, no PLA spots were observed when using antibodies against Fis1, a mitochondrial outer membrane protein facing the cytosol, or Hsp60, a mitochondrial matrix protein, suggesting that the distance between the cytosol and mitochondrial matrix is sufficient to prevent PLA signal (Fig. S1G). In contrast, PLA with antibodies for the confirmed partners MFF and Drp1 (Otera et al., 2010) yielded numerous spots, confirming the effectiveness of this technique (Fig. S1G).

PLA was then used to assess the colocalization of Dnm2 and EndoB1 under various autophagy-inducing conditions. We observed significant increases in PLA spots under each autophagy-inducing condition (Fig. 1E,F), which matched the colocalization observed by immunofluorescence and with fluorescently tagged proteins. We therefore conclude that the colocalization of Dnm2 and EndoB1 increases during autophagy.

### Colocalization of Dnm2 and EndoB1 with phagophores

Although the number of Dnm2-EndoB1 PLA spots increased when autophagy was induced, the number of Dnm2-EndoA1 PLA spots decreased, indicating a shift from endocytosis to autophagy (Fig. 1G). To further support a potential role in autophagy, we assessed colocalization with other autophagy proteins using PLA and fluorescent proteins. We observed a significant increase in colocalization of Dnm2-EndoB1 with EGFP-LC3 upon induction of autophagy (Fig. 1H,I). We then examined colocalization of PLA spots with several other autophagy-related proteins. Results, summarized in Fig. 1I, show additional increases in the fraction of Dnm2-EndoB1 PLA spots associated with two early autophagy markers (Atg2 and Fip200). No changes were seen in colocalization with Stx17 or Rab7, which are found on mature autophagosomes. There was a decrease in the fraction of PLA spots associated with LAMP1, which may indicate a shift away from the previously observed role in autophagolysosomal recycling (Schulze et al., 2013). We conclude that a large portion of Dnm2-EndoB1 PLA spots is located at or near sites of phagophore formation, but this association diminishes during later stages of autophagy.

### No effects of Dnm2 deletion on mitophagy or starvation-induced macroautophagy

Since Dnm2 and EndoB1 colocalize during autophagy induction, we tested whether these proteins contribute to the process. We first examined the effects of Dnm2 deletion on CCCP-induced mitophagy, as previous studies have linked Dnm2 to mitochondria (Karbowski et al., 2004; Lee et al., 2016). Super-resolution structured illumination microscopy (SIM) images of EGFP-LC3 and mitochondrial DsRed show cup-shaped phagophores surrounding the mitochondria in Dnm2 KO cells after 6 h of CCCP treatment (Fig. 2A). These phagophores appear similar to those in WT cells. Fluorescence images reveal CCCP-induced mitochondrial fragmentation in all cells, along with gradual mitochondrial removal over 24 h in both WT and Dnm2 cells (Fig. 2B). A similar pattern is observed in the western blot analysis of the mitochondrial matrix protein Hsp60 in Dnm2 KO cells (Fig. 2C,D), indicating that CCCP-driven Hsp60 degradation occurs over 24 h. If anything, degradation seems faster in Dnm2 KO. Uncropped western blots are shown in Fig. S6.

**Fig. 2.**
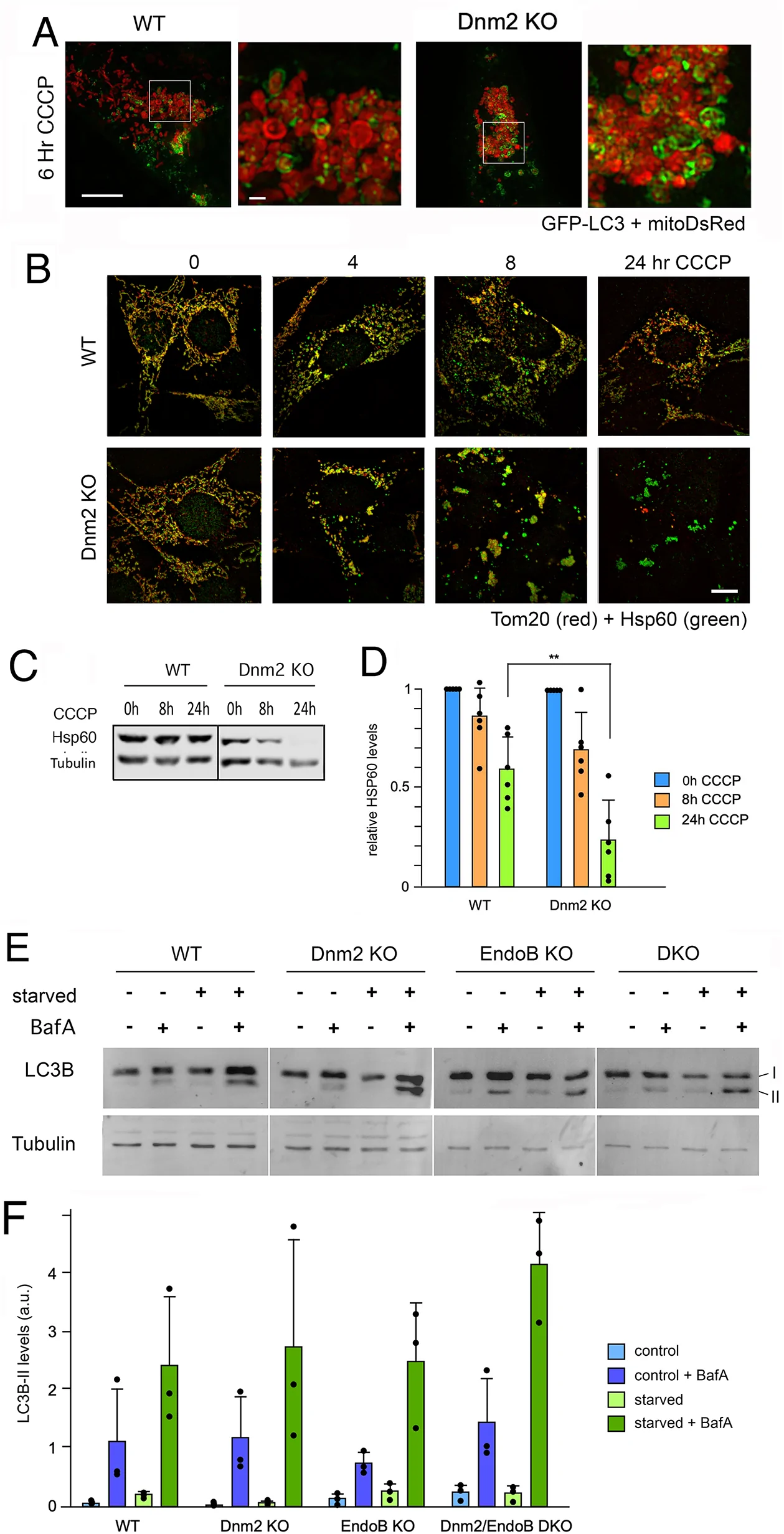
**Little effect of Dnm2 and EndoB1 deletions on mitophagy and macroautophagy.** (A) Super-resolution images (SIM) show the formation of LC3 membranes (green) encapsulating the mitochondria (red) in WT and Dnm2 KO MEFs treated with CCCP. Cells were treated for 6 h with 20 μM CCCP. Mitochondria were detected with mitoDsRed, and autophagic membranes were detected with EGFP-LC3. Scale bars: 10 μm for the overview and 1 μm for the enlargements. (B) Effects of CCCP on mitophagy in WT and Dnm2 KO MEFs observed with immunofluorescence microscopy of fixed cells using Tom20 (red) and Hsp60 (green) antibodies. At 24 h, most mitochondria are degraded. Scale bar: 10 μm. (C) Western blots showing the effects of CCCP on Hsp60 levels in WT and Dnm2 KO MEFs. (D) Average intensities of Hsp60 bands in four independent experiments, relative to tubulin levels and normalized to the 0-h time point. Average and s.d. are shown for six biological replicates. Statistical analysis used one-way ANOVA with Tukey's post hoc comparisons. (E) Western blots showing the effects of starvation-induced macroautophagy and bafilomycin A treatments on LC3B lipidation in WT, Dnm2, EndoB KO and DKO MEFs. (F) Average intensities of LC3B-II bands in four independent experiments, relative to tubulin levels, expressed in arbitrary units (a.u.). Average and s.d. are shown for three biological replicates. Statistical analysis used one-way ANOVA with Tukey's post hoc comparisons (**P<0.01).

To evaluate whether deletions of Dnm2 or EndoB affect macroautophagy, cells were starved for 4 h in Earle's balanced salt solution (EBSS) and then processed for cell extraction and western blot analysis of LC3B lipidation. The blots show no significant differences in LC3B-II levels among Dnm2, EndoB KO, or double KO (DKO) MEFs (Fig. 2E,F). Similar experiments with Dnm2 and EndoB KO HeLa cells also found no differences (Fig. S2A,B). We conclude that mitophagy and macroautophagy proceed normally in Dnm2 and EndoB KO cells.

### Transfer of LC3 to autophagolysosomes is unimpeded in Dnm2 KO cells

To monitor downstream events affected by loss of Dnm2 during autophagy, we transfected cells with RFP-GFP-LC3, which appears as yellow spots in the cytosol and red spots when autophagosomes fuse with lysosomes, as lysosomal acidification quenches GFP. Although the numbers of autophagosomes are similar (Fig. 3B), our results show that Dnm2 and EndoB KO cells exhibit more red fluorescence spots (autophagolysosomes) than WT cells after CCCP treatment (Fig. 3C). We conclude that autophagosomes in Dnm2 and EndoB KO cells move more quickly toward fusion with lysosomes compared to those in WT cells treated with CCCP. To further investigate the effects on autophagy, we measured the colocalization of LC3 and Rab7 after inducing autophagy with rapamycin. Colocalization of LC3 and Rab7 was not reduced in Dnm2 KO cells (Fig. 3D,E), indicating that autophagosome formation occurs normally in these cells.

**Fig. 3.**
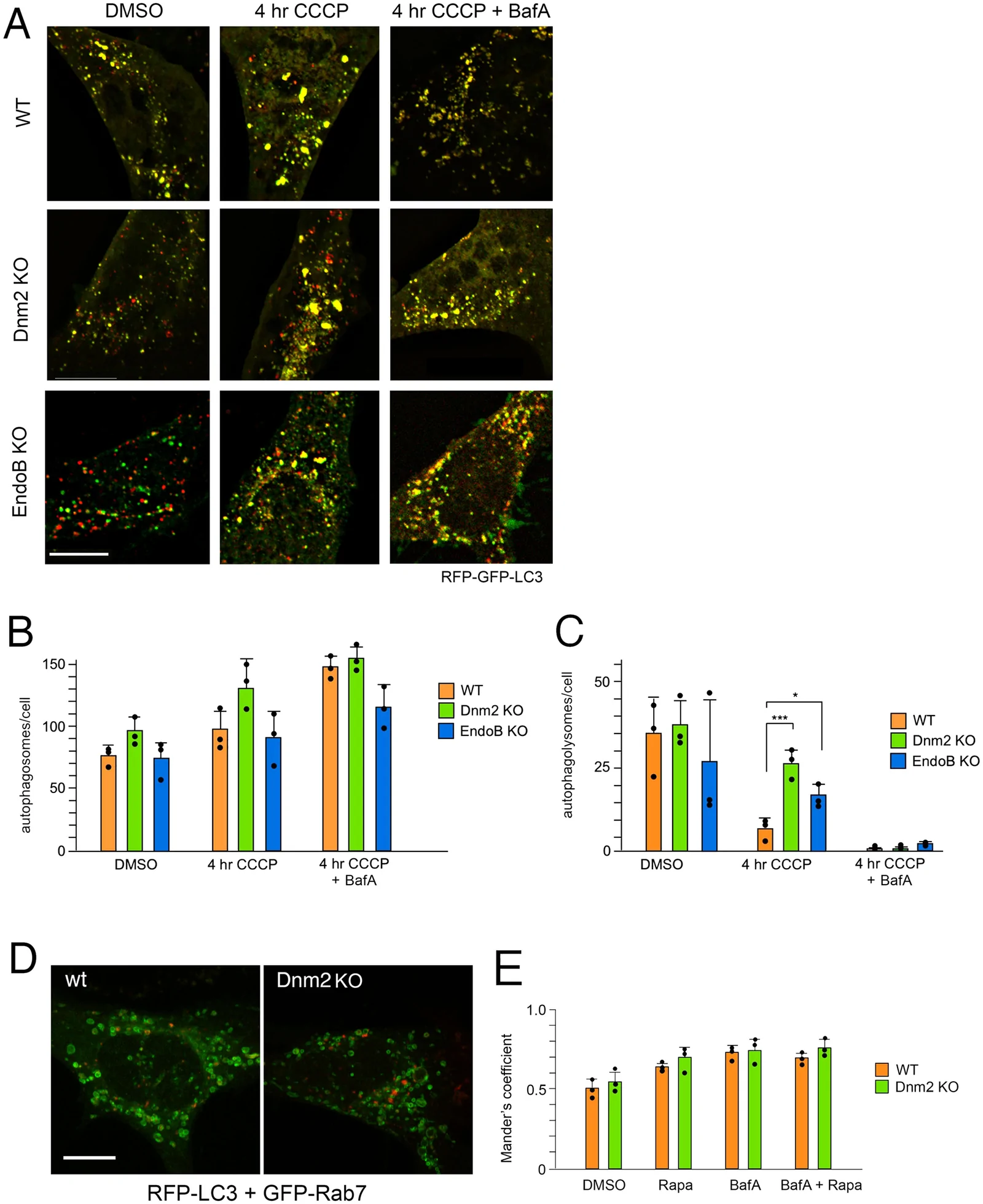
**Transfer of LC3 to lysosomes is unimpeded.** (A) Tracking autophagy with the RFP-GFP-LC3 marker in WT and in Dnm2 and EndoB KO MEFs shows transfer of LC3 to autophagolysosomes, visible as red spots due to acidic quenching of GFP. Scale bar: 10 μm. (B) Numbers of phagophores or autophagosomes per cell, detected as yellow spots, in WT and in Dnm2 and EndoB KO cells. Average and s.d. are shown for three biological replicates, each determined with five technical repeats. Statistical analysis used one-way ANOVA with Tukey's post hoc comparisons, but no significance was detected. (C) Numbers of autophagolysosomes, detected as red spots, are significantly increased in Dnm2 and EndoB KO MEFs treated with CCCP. Statistics as in panel B. (D) Live-cell imaging of the effects of WT and Dnm2 KO cells showing colocalization of RFP-LC3 and GFP-Rab7. In this case, autophagy was induced after 1-h treatment with rapamycin. Scale bar: 10 μm. (E) Levels of LC3 and Rab7 colocalization expressed as Manders' coefficient for untreated cells and cells treated with rapamycin, bafilomycin, or both. Average and s.d. are shown for three biological replicates, with seven technical replicates per biological replicate. Statistical analysis used one-way ANOVA with Tukey's post hoc comparisons, but no significance was detected (*P<0.05; ***P<0.001).

### Atg9A is retained on LC3-containing membranes in Dnm2 KO cells

The Atg9A protein, which helps deliver membrane to growing phagophores, is usually not incorporated into mature autophagosomes, suggesting that a recycling process retrieves this protein. We studied the potential effects of Dnm2 KO on the retrieval of Atg9A from autophagic membranes, as this process likely involves membrane scission in phagophores. We checked whether the retrieval blockage could be detected using fluorescent proteins. Dnm2 KO and WT cells were transfected with RFP-tagged Atg9A and EGFP-LC3 and then fixed for microscopy. We observed clear colocalization of Atg9A-myc and HA-LC3 by immunofluorescence (IF) in fixed Dnm2 KO cells, but not in WT cells (Fig. 4A), confirmed by Manders' coefficients (Fig. 4B). The effect of Dnm2 KO on colocalization was significantly reduced when cells were transfected with Dnm2-EGFP, which indicates that colocalization is not an off-target effect (Fig. S3A). Similar increases were observed in live Dnm2 KO and Dynasore-treated cells using RFP-Atg9A and GFP-LC3 (Fig. S3B,C). There is also a decrease in spots labeled only with Atg9A, but not LC3 (Fig. S3D), supporting the idea that fusion with phagophores reduces the supply of Atg9A vesicles. Changes in Atg9A and LC3 colocalization were also enhanced by treatment with bafilomycin A (Fig. S3E,F), in WT cells transfected with Dnm2 short interfering RNA (siRNA) (Fig. S3G-I), and in Dnm2 KO HeLa cells (Fig. S3J-L), confirming that Dnm2 is necessary to prevent Atg9A and LC3 from colocalizing in autophagosomes.

**Fig. 4.**
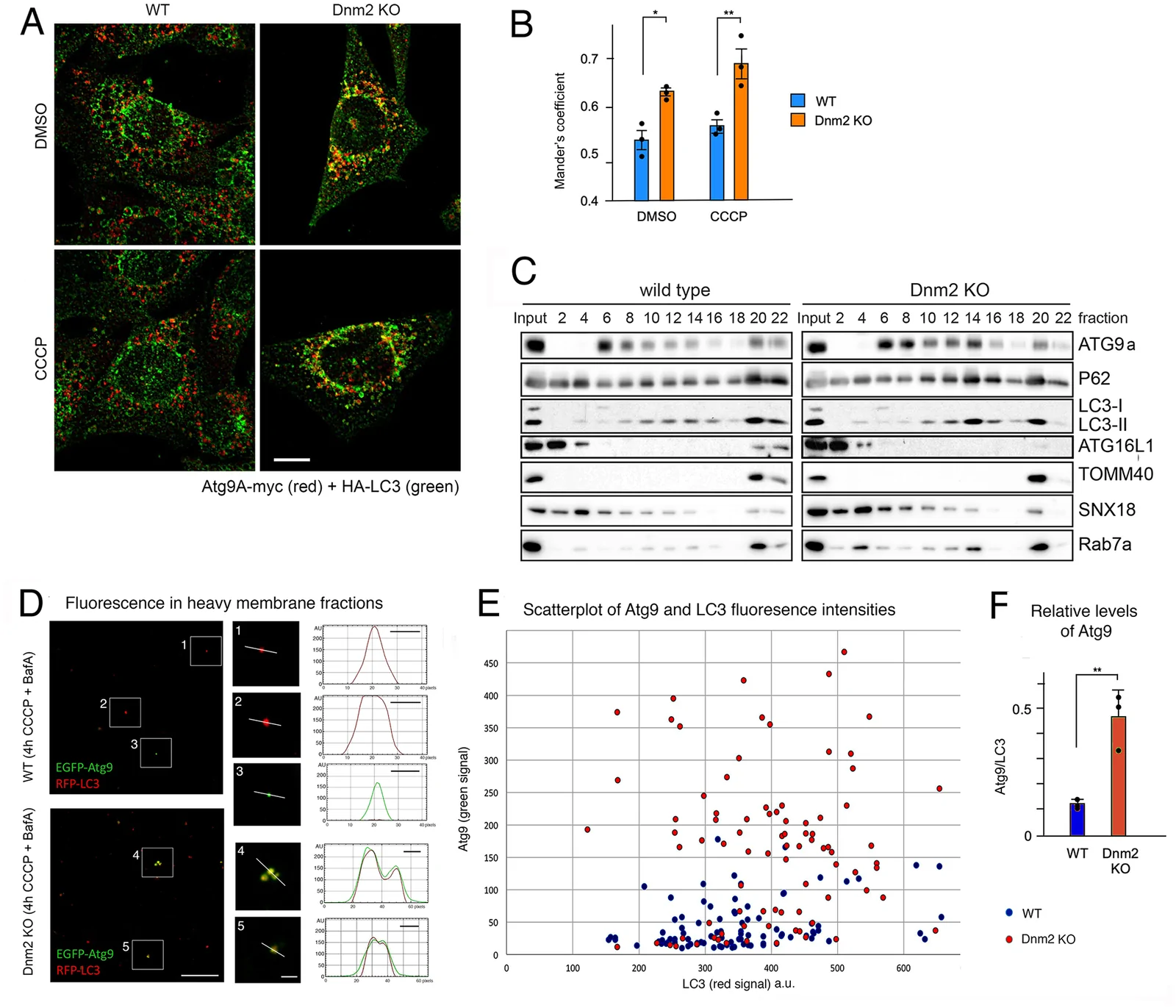
**Atg9A remains associated with LC3 in Dnm2 KO cells.** (A) Fixed cell fluorescence images showing stably expressed Atg9A-myc colocalizes with HA-LC3 in Dnm2 KO MEFs treated with CCCP, but not in WT MEFs. Scale bar: 10 μm. (B) Histogram showing Manders' coefficient for colocalization of Atg9A-myc and HA-LC3 in WT and Dnm2 KO MEFs after incubation with DMSO or CCCP. Average and s.d. are shown for three biological replicates, with five technical replicates per biological replicate. Statistical analysis used one-way ANOVA with Tukey's post hoc comparisons. (C) Subcellular fractionation of homogenates from WT and Dnm2 KO MEF cells, which were treated for 4 h with rapamycin. Membranous organelles were separated with a 1-22% Ficoll density gradient and analyzed with western blots. The bottom fractions (#20+22) contain mitochondria and other dense organelles such as mitophagosomes, autophagolysosomes, or lysosomes. Atg9A is normally present in light fractions (fraction #6) but partially shifts to an intermediate density (fraction #14) in Dnm2 KO cells, along with some p62, LC3-II and Rab7a. (D) Images of autophagosome-containing fractions from WT and Dnm2 KO cells transfected with EGFP-Atg9A and RFP-LC3, followed by 4-h treatments with CCCP and bafilomycin to induce mitophagy and to accumulate autophagosomes. Autophagosomes were enriched by differential centrifugation. Enlargements show individual spots with line tracings to plot fluorescence intensities. Scale bars: 10 μm in the overview images and 2 μm in the enlargements. The *x*-axis in the fluorescence intensity plots shows numbers of pixels (24 pixel/μm), and the bars show 500 nm. (E) Scatterplot of peak fluorescence intensities for individual spots, as shown in panel F (arbitrary units). (F) Histogram of the peak fluorescence intensities of Atg9A in LC3 spots relative to the peak intensities of Atg9A in spots without LC3. Average and s.d. are shown for three biological replicates with 30 technical replicates each. Statistical analysis used an unpaired Student's *t*-test (*P<0.05; **P<0.01).

Next, we used subcellular fractionation to detect potential abnormal Atg9A-containing organelles in Dnm2 KO cells. The distribution of these organelles was analyzed using Ficoll density gradients, as described previously (Orsi et al., 2012). Autophagy was induced in WT and Dnm2 KO MEF cells with rapamycin instead of CCCP because the mitophagosomes formed by CCCP could not be separated from intact mitochondria in Ficoll gradients. In contrast, rapamycin promotes the formation of less dense autophagosome-related structures that can be separated with Ficoll gradients. Atg9A is usually found in a light membrane fraction (fraction #6 in Fig. 4C), but some protein is detected in the heavy membrane fraction of induced cells (fraction #20). Other autophagy proteins, including p62 and LC3-II, are present in this fraction alongside mitochondrial (TOMM40) and endosomal/lysosomal (Rab7a) proteins. Importantly, Dnm2 KO cells show an additional buildup of certain proteins (Atg9A, p62, LC3-II, and Rab7a) in a new fraction (fraction #14 in Fig. 4C). This fraction does not contain Atg16L1, which acts early to promote LC3 lipidation, nor does it include SNX18, which was previously suggested to work with Dnm2 in Atg9A vesicle formation at early endosomes (Soreng et al., 2018). These findings suggest that fraction #14 contains abnormal autophagosomes with Atg9A, LC3-II, Rab7a, and p62. The increased presence of Rab7a suggests that this new fraction contains autophagolysosomes, which have not yet matured to form lysosomes. Their buoyancy may have been affected by the bafilomycin treatment, as this would have caused organelle swelling by increasing lysosomal pH. The presence of Atg9A in fraction #14 is significant because this protein is normally absent at a stage when Atg16L1 is removed, and autophagosomes have proceeded to fuse with Rab7a-containing compartments (Judith et al., 2019; Olivas et al., 2023; Orsi et al., 2012).

To further investigate whether Atg9A and LC3 coexist in the same compartments in Dnm2 KO cells, we imaged individual autophagosomes from crude biochemical isolates as described previously (Gao et al., 2010b). MEF cells were transfected with EGFP-Atg9A and RFP-LC3 and then treated for 4 h with CCCP and bafilomycin to induce mitophagy and promote autophagosome accumulation. These autophagosomes were enriched through differential centrifugation and washed twice in 1.0 M NaCl to dissociate vesicle clusters held together by ionic interactions. Fractions of these samples were mounted on slides and examined using fluorescence microscopy. Extracts from WT cells showed fluorescent spots containing LC3 but little to no Atg9A, likely representing mature autophagosomes (Fig. 4D). Occasionally, spots with Atg9A but lacking LC3 were observed, which may be Atg9A vesicles or organelles. In contrast, extracts from Dnm2 KO cells consistently displayed spots with overlapping LC3 and Atg9A fluorescence (Fig. 4D). Selected line tracings for these spots are shown in Fig. 4D, with a third example for Dnm2 KO cells shown in Fig. S4A-C. The LC3 and Atg9A fluorescence intensity tracings closely matched in Dnm2 KO extracts, indicating that membranes containing both proteins had fully merged. A scatterplot of peak intensities for various spots showed that many in Dnm2 KO extracts contain both proteins, whereas this was not the case in WT extracts (Fig. 4E). Additionally, a histogram of peak intensities from three independent experiments revealed significant differences in their distribution in Dnm2 KO cells (Fig. 4F).

We hypothesized that a Dnm2-dependent process could retrieve SNARE proteins along with Atg9A vesicles and that this retrieval would be impaired in Dnm2 KO cells. We examined the localization of VAMP7, a v-SNARE that is usually found in late endosomes but is also associated with Atg9A vesicles (Aoyagi et al., 2018; Yu et al., 2017). Our results show increased colocalization of VAMP7 with LC3 in Dnm2 KO cells, and this colocalization increases upon CCCP-induced mitophagy (Fig. S4D,E), suggesting that VAMP7 is also retrieved from phagophores with the help of Dnm2.

### Dnm2 promotes the retrieval of Atg9A from autophagosomes

We used live-cell imaging to explore whether Dnm2 localizes to sites of Atg9A retrieval from autophagosomes. We tracked RFP-tagged Dnm2 near or on phagophores labeled with BFP-LC3 and vesicles labeled with GFP-Atg9A. Even in untreated cells, we observed RFP-Dnm2 spots positioned between BFP-LC3-labeled phagophores and GFP-Atg9A-labeled vesicles. These Dnm2 spots disappeared within a second, and the organelles separated. Examples are shown in Fig. 5A. The frequency of these events was low (one in three videos for untreated cells), likely due to limitations of the imaging system (50 images of a cell segment in a single plane at 350-ms intervals). However, extrapolating to entire cells suggests a rough estimate of about 25 events per cell per minute, consistent with a role in autophagy. Due to photobleaching and rapid organelle movement, we could not determine if multiple fission events happen on growing phagophores. Fission at the plasma membrane can be blocked by the dynamin inhibitor Dynole 34-2 and promoted by the dynamin activator Ryngo 1-23 (Jackson et al., 2015; Lasic et al., 2017). We tested how these chemicals affected the appearance and disappearance of RFP-Dnm2 spots between BFP-LC3- and GFP-Atg9A-labeled organelles. Representative images of cells treated with Dynole 34-2 or Ryngo 1-23 are shown in Fig. S5A. We observed a significant decrease in Dnm2 spots with Dynole 34-2 and an increase with Ryngo 1-23 (Fig. 5B), supporting a role for Dnm2 during this stage of phagophore formation. Representative images are shown in Fig. S5A.

**Fig. 5.**
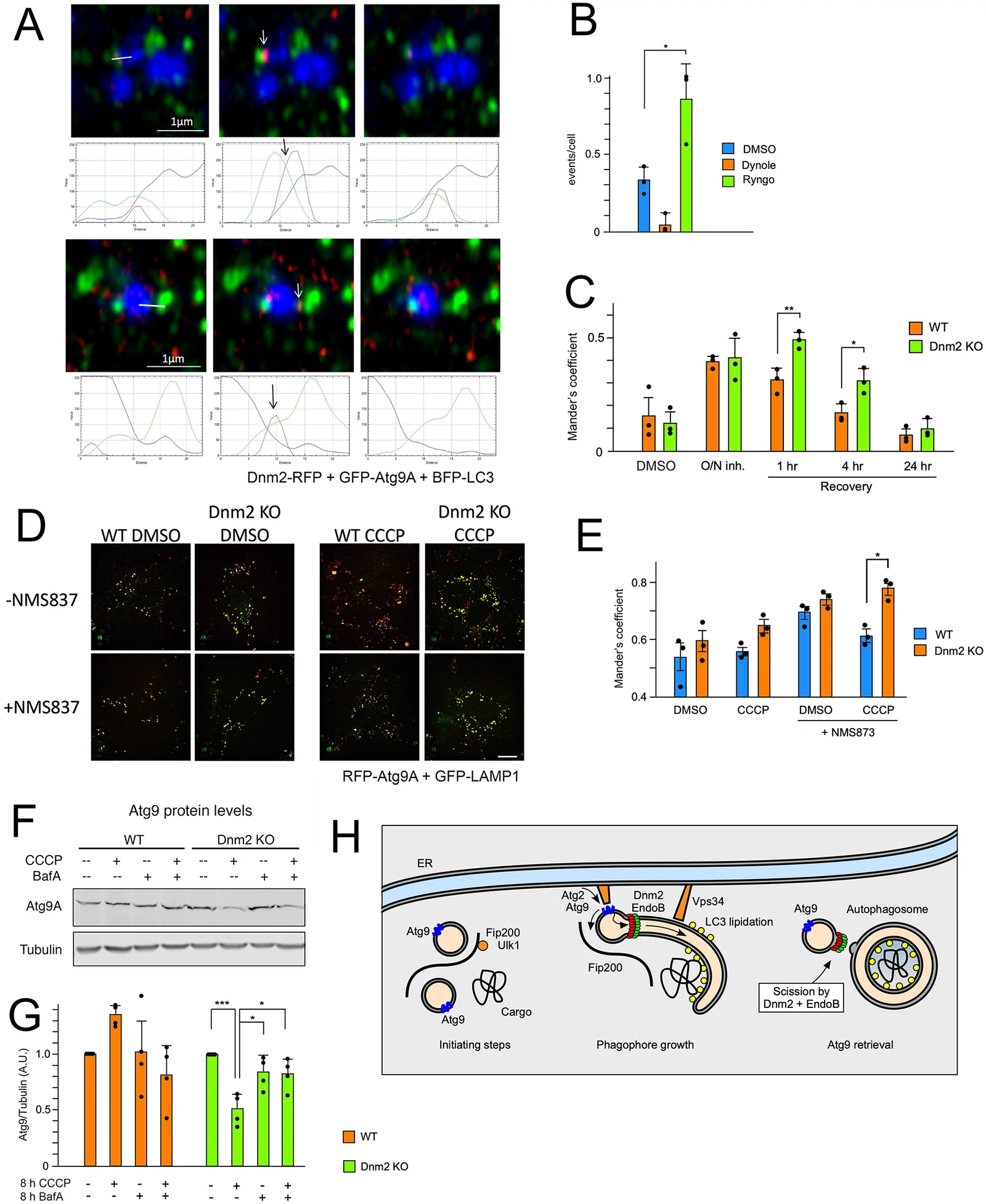
**Dnm2 promotes Atg9A retrieval from phagophores.** (A) Examples of Dnm2-RFP wedged between spots with GFP-Atg9A and BFP-LC3, with fluorescence intensities for the transect plotted below. Frames in live-cell imaging were separated by 350-400 ms. Scale bars: 1 μm. (B) Mean frequencies of Dnm2-RFP spots transiently wedged between GFP-Atg9A and BFP-LC3 spots in untreated cells and cells treated with Dynole 34-2 or Ryngo 1-23. The frequencies were low because only a small part of a cell was observed for a limited time (20 s), but their transient nature and the opposing effects of a dynamin inhibitor and an activator suggest a functional connection with fission events that separate Atg9A and LC3. Average and s.d. are shown for three biological replicates, each determined with four to seven technical repeats. Statistical analysis used one-way ANOVA with Dunnett's post hoc test across biological replicates. (C) Delayed dissociation of Atg9A and p62 in Dnm2 KO cells. A buildup of Atg9A and p62 was induced for 16 h with the Vps34 inhibitor Vps34-IN1. This was followed by inhibitor washout for the indicated durations. Association and dissociation of Atg9A and p62 were monitored by immunofluorescence microscopy of endogenous proteins in WT and Dnm2 KO HeLa cells. These cells were fixed with paraformaldehyde before imaging. Average and s.d. are shown for three biological replicates, each determined with five technical repeats. Statistical analysis used an unpaired Student's *t*-test on the biological replicates for each treatment. (D,E) Colocalization of RFP-Atg9A and GFP-LAMP1 after treatment of MEFs with CCCP is increased by adding the p97 inhibitor NMS-873. Cells were incubated for 4 h with DMSO or 10 μM NMS-873 and DMSO or 10 μM CCCP as indicated. Scale bar: 10 μm. Average and s.d. are shown for three biological replicates, each determined with five technical repeats. Statistical analysis used one-way ANOVA with Tukey's post hoc comparisons of the biological replicates for each treatment. (F,G) Western blot showing decreased amounts of Atg9A protein in CCCP-treated Dnm2 KO MEFs. The lower panel shows quantification of Atg9A protein levels in blots of WT and Dnm2 KO cells. Levels decrease in Dnm2 KO cells after treatment with CCCP, but this decrease is partially prevented by treatment with bafilomycin A, suggesting that Atg9A is degraded by autophagy in Dnm2 KO cells. Band intensities were determined with LI-COR software. Average and s.d. are shown for four biological replicates. Statistical analysis used one-way ANOVA with Tukey's post hoc comparisons of the biological replicates for each cell line. (H) Functions of Dnm2 and EndoB1 on phagophores. Autophagy is triggered by activation of ULK1 kinase, which is associated with the scaffold protein FIP200 and with Atg9A-containing vesicles. The phagophore then grows from the Atg9A vesicles by an Atg2-mediated lipid transfer from other organelles, such as the ER, and these lipids are equilibrated between the leaflets of the phagophore membrane by the scramblase function of Atg9A. Dnm2 and EndoB1 may have already assembled on this growing phagophore, as suggested by their colocalization with Atg2 and Fip200 (Fig. 1I). Vps34-mediated lipid phosphorylation and subsequent LC3 lipidation promote phagophore encapsulation of cargo. Once the phagophore is complete, EndoB1 and Dnm2 facilitate fission, forming a vesicle that retrieves Atg9A. For simplicity, only a subset of autophagy proteins is shown (*P<0.05; **P<0.01; ***P<0.001).

We used a pulse-chase approach to study the timing of Atg9A trafficking in the absence of Dnm2. LC3 lipidation was blocked for 16 h using the Vps34 inhibitor Vps34-IN1, followed by washout and recovery periods of increasing length. In these experiments, we used HeLa cells to track Atg9A and p62 colocalization by immunofluorescence, as Atg9A antibodies are more specific in human cells than in mouse cells. Both WT and Dnm2 KO cells showed increased colocalization of Atg9A and p62 after 16 h of Vps34-IN1 treatment (Fig. 5C), indicating clustering of Atg9A vesicles near stalled phagophores. This colocalization gradually decreased over 24 h after removing Vps34-IN1, but recovery was notably slower in Dnm2 KO cells (Fig. 5C), suggesting impaired Atg9A retrieval. Representative images are shown in Fig. S5B, and a western blot, shown in Fig. S5C, demonstrates that LC3B-II recovery is unaffected by the loss of Dnm2.

We also examined the colocalization of Atg9A and LAMP1, a lysosomal protein that is not typically associated with Atg9A vesicles. Cells were treated for 4 h with or without 10 μM p97 inhibitor NMS-873 to prevent the extraction of Atg9A from lysosomes and degradation by the proteasome, along with 10 μM CCCP to induce mitophagy. The results showed a significant increase in colocalization of Atg9A and LAMP1 in Dnm2 KO cells following CCCP induction (Fig. 5D,E). Similarly, enhanced colocalization was observed with pepstatin and E64d to prevent lysosomal degradation of Atg9A (Fig. S5D,E), suggesting that Atg9A on amphisomes can be internalized and then degraded in autophagolysosomes by internal proteases as well. The extraction of ubiquitinated Atg9A by p97 would act in parallel with this degradative pathway. We conclude that a greater proportion of Atg9A is degraded in DNM2 KO cells treated with CCCP than in WT cells.

Lastly, we used western blots to monitor Atg9A levels in Dnm2 KO MEFs. The Atg9A protein levels decrease when mitophagy is induced in Dnm2 KO cells with CCCP, but not in WT cells (Fig. 5F,G). This decrease is halted with bafilomycin, suggesting that Atg9A is degraded by lysosomal degradation in Dnm2 KO cells, but not in WT MEFs. We also noted that mutant and WT cells have similar Atg9A protein levels under basal conditions, suggesting that any additional Atg9A loss through basal autophagy in the mutant is small. Atg9A has a half-life of 23.8 h in HeLa cells (approximately 2.9% turnover per hour; comparable data are unavailable for MEFs) (Cambridge et al., 2011). Recent studies indicate that basal autophagy utilizes only a very small fraction of the large, cycling Atg9A pool in TGN, endosomal, and recycling membranes, potentially as little as one Atg9A vesicle per productive phagophore (Broadbent et al., 2023; Olivas et al., 2023). Thus, even if Atg9A is mistargeted and degraded in Dnm2 KO cells, the resulting additional turnover is likely to be small under basal conditions. Consistent with this interpretation, differences in Atg9A levels become apparent only when autophagic demand is substantially increased, such as during 8 h of CCCP-induced mitophagy. We therefore expect differences in Atg9A synthesis rates between mutant and WT cells to be very small under basal conditions.

Together, these experiments demonstrate that Atg9A retrieval from phagophores is impaired in Dnm2 KO cells and that Atg9A is subsequently degraded in lysosomes. Although the overall rates of autophagy do not decrease in Dnm2 KO cells, there may be other functional consequences because some early autophagy proteins, such as Atg9A and SNAREs, may be turned over more rapidly than those in WT cells, and their retention on phagophores could interfere with downstream processes. Collectively, our results suggest that Dnm2 and EndoB facilitate membrane fission to retrieve Atg9A from phagophores before autophagosome completion and fusion with lysosomes (Fig. 5H).

## DISCUSSION

Our data reveal an unexpected role for Dnm2 in autophagy. We find that Dnm2 facilitates the retrieval of Atg9A from growing phagophores, consistent with the previously reported absence of Atg9A from mature autophagosomes (Orsi et al., 2012). Our findings provide five lines of evidence demonstrating that Dnm2 is essential for this retrieval process. First, Dnm2 and EndoB colocalize with each other and with autophagy markers like LC3 during autophagy induction (Fig. 1). Second, mitophagy and starvation-induced macroautophagy proceed normally in cells lacking Dnm2 and EndoB (Figs 2 and 3). Third, we observe significant colocalization of Atg9A with LC3 in Dnm2 KO cells and in extracts upon inducing autophagy (Fig. 4). Additionally, SNARE proteins involved in the fusion of Atg9A vesicles with phagophores also merge with LC3 under these conditions in Dnm2 KO cells (Fig. S4). Similar effects are observed with other methods of Dnm2 impairment, such as siRNA knockdown (Fig. S3). These results suggest that transmembrane proteins in Atg9A vesicles colocalize with LC3-containing membranes when Dnm2 function is disrupted. Fourth, Dnm2 appears in transient spots between Atg9A- and LC3-positive compartments, and chemical inhibitors or activators of Dnm2 modulate the number of these spots in live cells (Fig. 5A,B). Fifth, the number of Atg9A vesicles decreases when autophagy is induced in Dnm2 KO cells, indicating that Atg9A vesicles fail to reform after seeding phagophores (Fig. S3B,D). Moreover, Atg9A protein levels are reduced in Dnm2 KO cells and decrease further upon autophagy induction (Fig. 5F,G). This reduction is prevented by bafilomycin, suggesting that Atg9A is incorporated into autophagosomes and degraded via autophagy when Dnm2 function is impaired. Overall, these findings show that Dnm2 is necessary for recycling transmembrane proteins from phagophores.

The new role of Dnm2 and EndoB in retrieving Atg9A from phagophores appears to conflict with previous ideas where Dnm2 separates Atg9A-containing vesicles from recycling endosomes, either with EndoB1 (Takahashi et al., 2016) or with SNX18 (Soreng et al., 2018) during starvation. In those cases, loss of Dnm2 or EndoB1 would prevent Atg9A from reaching the sites of phagophore formation, causing it to accumulate in other compartments, which would contradict our findings. Different origins of phagophores could explain some of these differences. For instance, it has been shown that phagophores can grow from recycling endosomes (Puri et al., 2018), while earlier research suggested that they originate from or near the ER (Karanasios et al., 2016; Orsi et al., 2012). The ER proximity explanation aligns with the newly discovered roles of Atg2 and Atg9A in expanding phagophores from Atg9A vesicles close to the ER or other organelles (Chang et al., 2021; Noda, 2021), and it supports a role for Dnm2 in retrieving Atg9A from those phagophores.

Dnm2 and EndoB1 may also act as barriers to prevent Atg9A from dispersing along expanding phagophores, while still allowing lipids to flow freely from transfer sites with Atg2 and Atg9A to the rest of the phagophore. Interestingly, this function appears to be absent in yeast, where Atg9A is transported to the vacuole and then retrieved (Yamamoto et al., 2012). Other aspects of the fission process have not yet been characterized, but some similarities can be observed with Dnm2-driven fission events at the plasma membrane. In addition to Dnm2's well-known roles in clathrin- and caveolin-dependent endocytosis (Henley et al., 1998; van der Bliek et al., 1993) and clathrin-/caveolin-independent endocytosis (Conner and Schmid, 2003), Dnm2 also facilitates fast endocytosis alongside endophilin A (FEME) (Renard et al., 2015). Whether EndoB1 and Dnm2 have roles in autophagy similar to Dnm2 and endophilin A in FEME remains unclear. However, the effects of the dynamin activator Ryngo 1-23 and the inhibitor Dynole 34-2 on the appearance of Dnm2 spots between Atg9A and LC3 membranes (Fig. 5B), as well as the impact of Dnm2 loss of function on Atg9A retrieval, support a role for Dnm2 in this process (Fig. 5H).

Our density fractionation experiment showed an accumulation of Atg9 in a novel fraction in Dmnmk2 KO cells (Fig. 4C). This fraction also contains p62, LC3-II, and Rab7, which suggests that it contains immature autophagolysosome intermediates. WT cells also have a small amount of LC3 and Rab7 in this fraction, but no Atg9. The increased levels of p62, LC3-II, Rab7, and especially Atg9 in fraction 14 suggest that Dnm2 KO delays the maturation of these autophagolysosome intermediates into mature lysosomes. Critically, however, Atg9 is not normally present in autophagolysosomes (Judith et al., 2019; Olivas et al., 2023; Orsi et al., 2012), even though Dnm2 may affect the maturation of autophagolysosomes as well.

In conclusion, our data suggest a model where Dnm2 facilitates Atg9A retrieval from expanding phagophores. Other possible roles in related processes, such as vesicle budding from endosomes (Soreng et al., 2018; Takahashi et al., 2016) or retrieval from autolysosomes (Fang et al., 2016; Schulze et al., 2013), as well as indirect effects from different vesicular transport pathways, are also plausible. However, our findings are most straightforwardly interpreted as direct effects on the retrieval of Atg9A-containing vesicles.

## MATERIALS AND METHODS

### Plasmids

The pMito-DsRed2 plasmid was obtained from Clontech. Addgene provided pcDNA3-mRuby2 (#40260), GFP-C1-PLCdelta-PH (#21179), Dnm2-EGFP (#34686), Dnm2-mCherryN1 (#27689), ptfLC3 (#21074), pMXs-puro-RFP-ATG9A (#60609), pEGFP-LC3 (# 24920), GFP-Rab7A (#61803), mTaqBFP2-ER-5 (#55294), pcDNA3-mRuby2 (#40260), pEGFP-C1-hAtg2A (#36456), pMXs-IP-EGFP-hFIP200 (#38192), pEGFP-VAMP7 (#42316), pmRFP-LC3 (#21075) and pMRXIP GFP-Stx17 (#45909). X. Soriano (Department of Cell Biology, University of Barcelona, Barcelona, Spain) provided the GFP-Bax plasmid. E. C. Dell'Angelica (Department of Human Genetics, UCLA School of Medicine, Los Angeles, CA, USA) provided the GFP-LAMP1 plasmid. To generate EndophilinB1-mRuby2, endophilin B1 coding sequences from cDNA were fused in frame to mRuby2 by PCR cloning into pcDNA3-mRuby2. EGFP-Atg9A, Dnm2-RFP and mTaqBFP2-LC3 constructs were generated using the same method with coding sequences from pMXs-puro-RFP-ATG9A, Dnm2-EGFP and mTaqBFP2-ER-5, respectively.

### Cell culture, transfection, gene KO and chemical treatments

WT HeLa cells were obtained from James Wohlschlegel (Department of Biological Chemistry, UCLA, Los Angeles, CA, USA), WT MEFs were obtained from David Chan (Department of Biology, Caltech, Pasadena, CA, USA), and MEFs with stable expression of FLAG-Parkin were obtained from Lars Dreier (UCLA, Los Angeles, CA, USA) (Sun et al., 2012). All cell lines were periodically checked for mycoplasma. MEFs were grown in DMEM with 10% FBS. Transient transfections were performed using jetPRIME according to the manufacturer's instructions (Polyplus). For siRNA, cells were grown in 6 cm dishes, transfected with 50 nM oligonucleotides using RNAiMAX (Invitrogen) and analyzed 72 h later. Deletions in the Dnm2 and EndoB1 genes were introduced into MEFs with FLAG-Parkin using CRISPR/Cas9 methods, as described previously (Ran et al., 2013). Target sites were identified using the Boutros Lab website (https://www.e-crisp.org/). The Dnm2 guide RNA (gRNA) sequence was 5′-GATGGCAAACACGTGCTTGT-3′. The EndoB1 gRNAs were 5′-GCAGGAACTGAGTTTGGCCC-3′ and 5′-GGATTTCAACGTGAAGAAGC-3′. The gRNAs were cloned into the px459 plasmid, containing puromycin resistance and Cas9 genes, and 1.2 μg of the plasmid was transfected into MEF cells, followed by 24 h of puromycin selection. Surviving colonies were isolated, genotyped and analyzed using western blots. HeLa cells were also grown in DMEM with 10% FBS. The gRNA used to knock out Dnm2 in HeLa cells was 5′-ACAGGGGCCGGCCCTGGACC-3′. The KO procedure was the same as that described for MEF cells.

Since the EndoB1 KO cells lacked Parkin expression, they were stably transfected with an EF1alpha-promoter-driven Parkin expression construct in a PiggyBac vector. This construct was transfected into EndoB KO MEFs along with the PBase vector from VectorBuilder at equal concentrations (2 μg per well in a six-well plate). Atg9A-myc and HA-LC3B constructs were similarly stably transfected using the PiggyBac protocol. At 24 h post-transfection, the cells were subjected to puromycin selection at increasing concentrations ranging from 1 to 5 μg/ml for 1-2 days. After selection, surviving colonies were isolated with cloning rings, expanded and analyzed for Parkin expression levels by western blotting. A clone exhibiting Parkin expression levels consistent with those observed in WT cells expressing FLAG-Parkin and in Dnm2 cells expressing FLAG-Parkin was selected for further analysis.

Amino acid starvation was induced by washing the cells twice with EBSS containing calcium and magnesium (Thermo Fisher Scientific) and incubating them for 4 h. The following chemicals, along with their respective final concentrations, were obtained from Sigma-Aldrich: 20 μM CCCP, 40 μg/ml antimycin A, 10 μM actinomycin D and 1 μM 4-hydroxytamoxifen. The following chemicals, along with their respective final concentrations, were obtained from InvivoGen: 0.5 μM bafilomycin A1 and 10 μM rapamycin. Staurosporine (Thermo Fisher Scientific) was used at 2 μM. Z-VAD-FMK (BD Bioscience) was used at 20 μM. Dynasore (EMD Millipore) was used at 80 μM. VPS34-IN1 (Selleck Chemicals) was used at 10 μM. NMS-873 (Sigma-Aldrich) was used at 10 μM. Pepstatin and E64d (both from Sigma-Aldrich) were used at 5 μg/ml.

### Immunoblotting, immunofluorescence, and PLA

Total cell lysates for western blots were prepared using RIPA buffer. The samples were subjected to SDS-PAGE and transferred to nitrocellulose or polyvinylidene difluoride (PVDF) membranes. The membranes were blocked with Odyssey Blocking Buffer (LI-COR) and incubated overnight at 4°C with primary antibodies. The membranes were then washed with PBS-T and incubated with IRDye 800CW or 670RD secondary antibodies (LI-COR). Fluorescent bands were detected using an Odyssey scanner and analyzed using Image Studio software (LI-COR). For immunofluorescence images, cells were grown on 12 mm coverslips, fixed for 10 min with 4% paraformaldehyde in PBS, permeabilized for 5 min with 0.1% Triton X-100 in PBS, blocked for 1 h with goat or donkey serum in PBS-T and incubated with primary antibodies. One exception was the immunofluorescence of Dnm2 (Fig. 1A,B), for which the cells were first permeabilized with 0.05% digitonin to remove soluble cytosolic proteins (Liu et al., 1998), before fixation with 4% paraformaldehyde. Secondary antibodies were Alexa-Fluor-488-, 594- or 647-conjugated goat anti-mouse or rabbit IgG (Invitrogen). PLAs were conducted with Duolink as recommended by the manufacturer (Sigma-Aldrich). Cells expressing fluorescent proteins were also fixed with 4% paraformaldehyde, except when live cells were imaged, as indicated in the figure legends.

### Antibodies

Rabbit anti-endophilin A1, mouse anti-SQSTM1 (p62), rabbit anti-Dnm2, mouse anti-Hsp60, rabbit anti-cytochrome c and mouse anti-VDAC1/Porin were obtained from Abcam. Rabbit anti-LC3B and mouse anti-tubulin antibodies were obtained from Sigma-Aldrich. Goat anti-Dnm2 and rabbit anti-Tom20 antibodies were obtained from Santa Cruz Biotechnology. Mouse anti-Tim23 and mouse anti-Drp1 antibodies were obtained from BD Biosciences. Rabbit anti-RAB7A antibody was obtained from Proteintech. Mouse anti-endophilin B1 antibody was obtained from Imgenex. Rabbit anti-SNX18 and rabbit anti-ATG9A antibodies were obtained from GeneTex. See Table S1 for a list of antibodies, their sources and catalogue numbers.

### Microscopy

Standard immunofluorescence and live-cell images were obtained using a Marianas spinning disk confocal system from Intelligent Imaging Innovations, which uses an Axiovert microscope (Carl Zeiss Microscopy) with 40×/1.4 and 100×/1.4 oil objectives, a CSU22 spinning disk (Yokogawa), an Evolve 512 Electron Multiplying Charge-Coupled Device (EMCCD) camera (Photometrics) and a temperature control unit (Okolab). In experiments where live-cell imaging was conducted, the cells were cultured in glass-bottom dishes (Mattek) and imaged *in situ* at 37°C. Super-resolution images were acquired using SIM with a DeltaVision OMX SR (General Electric). Fiji ImageJ software was used for image analysis. Time-lapse images of live cells were acquired with a Zeiss Airyscan microscope equipped with a Fast-Airyscan module and processed with the Airyscan software.

### Subcellular fractionation

Extracts for imaging of partially purified autophagosomes were essentially made as described previously (Gao et al., 2010b). In brief, MEF cells were transfected with EGFP-Atg9A and RFP-LC3. After 16 h, the cells were treated with 20 μM CCCP and 0.5 μM bafilomycin for 4 h. The cells were harvested in mitochondrial isolation buffer (0.25 M sucrose, 1 mM EDTA, and 20 mM HEPES at pH 7.4) on ice and disrupted by passing them 20 times through a 22 G needle. Nuclei and unbroken cells were then removed by low-speed centrifugation (10 min at 600 ***g***) followed by enrichment of autophagosomes by medium-speed centrifugation (20 min at 10,000 ***g***). The resulting fractions were washed twice in 1.0 M NaCl in mitochondrial isolation buffer containing a protease inhibitor to dissociate clusters of vesicles that were potentially held together by ionic interactions. Samples of the resulting fractions were mounted on slides and examined by fluorescence microscopy.

For Ficoll gradient analysis, WT and Dnm2 KO MEF cells were treated with 0.5 mM bafilomycin A for 4 h, followed by a 2-h treatment with 0.5 mM bafilomycin A and 5 μM rapamycin to induce autophagy. The cells were then fractionated as described previously (Orsi et al., 2012), with a few modifications. In brief, cells harvested from four confluent 15 cm dishes were washed, scraped, and resuspended in homogenization buffer containing 250 mM sucrose, 20 mM HEPES/KOH (pH 7.4) and 0.5 mM PMSF, followed by homogenization with 15 passes through a 25 G needle. Nuclei and unbroken cells were removed by low-speed centrifugation. The equivalent of 2 mg protein was loaded onto a 1-22% Ficoll gradient, with a total volume of 10 ml layered onto a 1 ml 45% Nycodenz cushion in homogenization buffer. The preparations were then centrifuged in a Beckmann SW41 rotor for 14.5 h at 20,000 rpm at 4°C. Twenty fractions of 0.5 ml were collected, and the even-numbered fractions were analyzed by western blots using 40 μl samples.

## Supplementary Material



10.1242/biolopen.062632_sup1 Supplementary information

## References

[BIO062632C1] Achiriloaie, M., Barylko, B. and Albanesi, J. P. (1999). Essential role of the dynamin pleckstrin homology domain in receptor-mediated endocytosis. *Mol. Cell. Biol.* 19, 1410-1415. 10.1128/MCB.19.2.1410 PMC116069 9891074

[BIO062632C2] Antonny, B., Burd, C., De Camilli, P., Chen, E., Daumke, O., Faelber, K., Ford, M., Frolov, V. A., Frost, A., Hinshaw, J. E. et al. (2016). Membrane fission by dynamin: what we know and what we need to know. *EMBO J.* 35, 2270-2284. 10.15252/embj.201694613 PMC5090216 27670760

[BIO062632C3] Aoyagi, K., Itakura, M., Fukutomi, T., Nishiwaki, C., Nakamichi, Y., Torii, S., Makiyama, T., Harada, A. and Ohara-Imaizumi, M. (2018). VAMP7 regulates autophagosome formation by supporting Atg9a functions in pancreatic beta-cells from male mice. *Endocrinology* 159, 3674-3688. 10.1210/en.2018-00447 30215699

[BIO062632C4] Bento, C. F., Renna, M., Ghislat, G., Puri, C., Ashkenazi, A., Vicinanza, M., Menzies, F. M. and Rubinsztein, D. C. (2016). Mammalian autophagy: how does it work? *Annu. Rev. Biochem.* 85, 685-713. 10.1146/annurev-biochem-060815-014556 26865532

[BIO062632C5] Broadbent, D. G., Barnaba, C., Perez, G. I. and Schmidt, J. C. (2023). Quantitative analysis of autophagy reveals the role of ATG9 and ATG2 in autophagosome formation. *J. Cell Biol.* 222, e202210078. 10.1083/jcb.202210078 PMC10148237 37115157

[BIO062632C6] Cambridge, S. B., Gnad, F., Nguyen, C., Bermejo, J. L., Kruger, M. and Mann, M. (2011). Systems-wide proteomic analysis in mammalian cells reveals conserved, functional protein turnover. *J. Proteome Res.* 10, 5275-5284. 10.1021/pr101183k 22050367

[BIO062632C7] Chang, C., Jensen, L. E. and Hurley, J. H. (2021). Autophagosome biogenesis comes out of the black box. *Nat. Cell Biol.* 23, 450-456. 10.1038/s41556-021-00669-y PMC8122082 33903736

[BIO062632C8] Chappie, J. S., Acharya, S., Leonard, M., Schmid, S. L. and Dyda, F. (2010). G domain dimerization controls dynamin's assembly-stimulated GTPase activity. *Nature* 465, 435-440. 10.1038/nature09032 PMC2879890 20428113

[BIO062632C9] Choi, A. M., Ryter, S. W. and Levine, B. (2013). Autophagy in human health and disease. *N Engl. J. Med.* 368, 1845-1846. 10.1056/NEJMra1205406 23656658

[BIO062632C10] Conner, S. D. and Schmid, S. L. (2003). Regulated portals of entry into the cell. *Nature* 422, 37-44. 10.1038/nature01451 12621426

[BIO062632C11] Faelber, K., Posor, Y., Gao, S., Held, M., Roske, Y., Schulze, D., Haucke, V., Noe, F. and Daumke, O. (2011). Crystal structure of nucleotide-free dynamin. *Nature* 477, 556-560. 10.1038/nature10369 21927000

[BIO062632C12] Fang, X., Zhou, J., Liu, W., Duan, X., Gala, U., Sandoval, H., Jaiswal, M. and Tong, C. (2016). Dynamin regulates autophagy by modulating lysosomal function. *J. Genet. Genomics* 43, 77-86. 10.1016/j.jgg.2015.10.005 26924690

[BIO062632C13] Farsad, K., Ringstad, N., Takei, K., Floyd, S. R., Rose, K. and De Camilli, P. (2001). Generation of high curvature membranes mediated by direct endophilin bilayer interactions. *J. Cell Biol.* 155, 193-200. 10.1083/jcb.200107075 PMC2198845 11604418

[BIO062632C14] Ferguson, S. M. and De Camilli, P. (2012). Dynamin, a membrane-remodelling GTPase. *Nat. Rev. Mol. Cell Biol.* 13, 75-88. 10.1038/nrm3266 PMC3519936 22233676

[BIO062632C15] Ford, M. G., Jenni, S. and Nunnari, J. (2011). The crystal structure of dynamin. *Nature* 477, 561-566. 10.1038/nature10441 PMC4075756 21927001

[BIO062632C16] Gao, S., von der Malsburg, A., Paeschke, S., Behlke, J., Haller, O., Kochs, G. and Daumke, O. (2010a). Structural basis of oligomerization in the stalk region of dynamin-like MxA. *Nature* 465, 502-506. 10.1038/nature08972 20428112

[BIO062632C17] Gao, W., Kang, J. H., Liao, Y., Ding, W. X., Gambotto, A. A., Watkins, S. C., Liu, Y. J., Stolz, D. B. and Yin, X. M. (2010b). Biochemical isolation and characterization of the tubulovesicular LC3-positive autophagosomal compartment. *J. Biol. Chem.* 285, 1371-1383. 10.1074/jbc.M109.054197 PMC2801263 19910472

[BIO062632C18] Gomez-Sanchez, R., Rose, J., Guimaraes, R., Mari, M., Papinski, D., Rieter, E., Geerts, W. J., Hardenberg, R., Kraft, C., Ungermann, C. et al. (2018). Atg9 establishes Atg2-dependent contact sites between the endoplasmic reticulum and phagophores. *J. Cell Biol.* 217, 2743-2763. 10.1083/jcb.201710116 PMC6080931 29848619

[BIO062632C19] Guardia, C. M., Tan, X. F., Lian, T., Rana, M. S., Zhou, W., Christenson, E. T., Lowry, A. J., Faraldo-Gomez, J. D., Bonifacino, J. S., Jiang, J. et al. (2020). Structure of human ATG9A, the only transmembrane protein of the core autophagy machinery. *Cell Rep.* 31, 107837. 10.1016/j.celrep.2020.107837 PMC7388177 32610138

[BIO062632C20] Henley, J. R., Krueger, E. W., Oswald, B. J. and McNiven, M. A. (1998). Dynamin-mediated internalization of caveolae. *J. Cell Biol.* 141, 85-99. 10.1083/jcb.141.1.85 PMC2132718 9531550

[BIO062632C21] Hurley, J. H. and Young, L. N. (2017). Mechanisms of autophagy initiation. *Annu. Rev. Biochem.* 86, 225-244. 10.1146/annurev-biochem-061516-044820 PMC5604869 28301741

[BIO062632C22] Jackson, J., Papadopulos, A., Meunier, F. A., McCluskey, A., Robinson, P. J. and Keating, D. J. (2015). Small molecules demonstrate the role of dynamin as a bi-directional regulator of the exocytosis fusion pore and vesicle release. *Mol. Psychiatry* 20, 810-819. 10.1038/mp.2015.56 25939402

[BIO062632C23] Jones, S. M., Howell, K. E., Henley, J. R., Cao, H. and McNiven, M. A. (1998). Role of dynamin in the formation of transport vesicles from the trans-Golgi network. *Science* 279, 573-577. 10.1126/science.279.5350.573 9438853

[BIO062632C24] Judith, D., Jefferies, H. B. J., Boeing, S., Frith, D., Snijders, A. P. and Tooze, S. A. (2019). ATG9A shapes the forming autophagosome through Arfaptin 2 and phosphatidylinositol 4-kinase IIIbeta. *J. Cell Biol.* 218, 1634-1652. 10.1083/jcb.201901115 PMC6504893 30917996

[BIO062632C25] Karanasios, E., Walker, S. A., Okkenhaug, H., Manifava, M., Hummel, E., Zimmermann, H., Ahmed, Q., Domart, M. C., Collinson, L. and Ktistakis, N. T. (2016). Autophagy initiation by ULK complex assembly on ER tubulovesicular regions marked by ATG9 vesicles. *Nat. Commun.* 7, 12420. 10.1038/ncomms12420 PMC4987534 27510922

[BIO062632C26] Karbowski, M., Jeong, S. Y. and Youle, R. J. (2004). Endophilin B1 is required for the maintenance of mitochondrial morphology. *J. Cell Biol.* 166, 1027-1039. 10.1083/jcb.200407046 PMC2172012 15452144

[BIO062632C27] Lasic, E., Stenovec, M., Kreft, M., Robinson, P. J. and Zorec, R. (2017). Dynamin regulates the fusion pore of endo- and exocytotic vesicles as revealed by membrane capacitance measurements. *Biochim. Biophys. Acta* 1861, 2293-2303. 10.1016/j.bbagen.2017.06.022 28669852

[BIO062632C28] Lee, E. and De Camilli, P. (2002). Dynamin at actin tails. *Proc. Natl. Acad. Sci. USA* 99, 161-166. 10.1073/pnas.012607799 PMC117532 11782545

[BIO062632C29] Lee, E., Marcucci, M., Daniell, L., Pypaert, M., Weisz, O. A., Ochoa, G. C., Farsad, K., Wenk, M. R. and De Camilli, P. (2002). Amphiphysin 2 (Bin1) and T-tubule biogenesis in muscle. *Science* 297, 1193-1196. 10.1126/science.1071362 12183633

[BIO062632C30] Lee, J. E., Westrate, L. M., Wu, H., Page, C. and Voeltz, G. K. (2016). Multiple dynamin family members collaborate to drive mitochondrial division. *Nature* 540, 139-143. 10.1038/nature20555 PMC5656044 27798601

[BIO062632C31] Levine, B. and Klionsky, D. J. (2017). Autophagy wins the 2016 Nobel prize in physiology or medicine: breakthroughs in baker's yeast fuel advances in biomedical research. *Proc. Natl. Acad. Sci. USA* 114, 201-205. 10.1073/pnas.1619876114 PMC5240711 28039434

[BIO062632C32] Liu, S. H., Marks, M. S. and Brodsky, F. M. (1998). A dominant-negative clathrin mutant differentially affects trafficking of molecules with distinct sorting motifs in the class II major histocompatibility complex (MHC) pathway. *J. Cell Biol.* 140, 1023-1037. 10.1083/jcb.140.5.1023 PMC2132696 9490717

[BIO062632C33] Liu, Y. W., Surka, M. C., Schroeter, T., Lukiyanchuk, V. and Schmid, S. L. (2008). Isoform and splice-variant specific functions of dynamin-2 revealed by analysis of conditional knock-out cells. *Mol. Biol. Cell* 19, 5347-5359. 10.1091/mbc.e08-08-0890 PMC2592655 18923138

[BIO062632C34] Maeda, S., Otomo, C. and Otomo, T. (2019). The autophagic membrane tether ATG2A transfers lipids between membranes. *eLife* 8, e45777. 10.7554/eLife.45777 PMC6625793 31271352

[BIO062632C35] Maeda, S., Yamamoto, H., Kinch, L. N., Garza, C. M., Takahashi, S., Otomo, C., Grishin, N. V., Forli, S., Mizushima, N. and Otomo, T. (2020). Structure, lipid scrambling activity and role in autophagosome formation of ATG9A. *Nat. Struct. Mol. Biol.* 27, 1194-1201. 10.1038/s41594-020-00520-2 PMC7718406 33106659

[BIO062632C36] Matoba, K., Kotani, T., Tsutsumi, A., Tsuji, T., Mori, T., Noshiro, D., Sugita, Y., Nomura, N., Iwata, S., Ohsumi, Y. et al. (2020). Atg9 is a lipid scramblase that mediates autophagosomal membrane expansion. *Nat. Struct. Mol. Biol.* 27, 1185-1193. 10.1038/s41594-020-00518-w 33106658

[BIO062632C37] Meinecke, M., Boucrot, E., Camdere, G., Hon, W. C., Mittal, R. and McMahon, H. T. (2013). Cooperative recruitment of dynamin and BIN/amphiphysin/Rvs (BAR) domain-containing proteins leads to GTP-dependent membrane scission. *J. Biol. Chem.* 288, 6651-6661. 10.1074/jbc.M112.444869 PMC3585104 23297414

[BIO062632C38] Noda, N. N. (2021). Atg2 and Atg9: intermembrane and interleaflet lipid transporters driving autophagy. *Biochim. Biophys. Acta Mol. Cell Biol. Lipids* 1866, 158956. 10.1016/j.bbalip.2021.158956 33932584

[BIO062632C39] Olivas, T. J., Wu, Y., Yu, S., Luan, L., Choi, P., Guinn, E. D., Nag, S., De Camilli, P. V., Gupta, K. and Melia, T. J. (2023). ATG9 vesicles comprise the seed membrane of mammalian autophagosomes. *J. Cell Biol.* 222, e202208088. 10.1083/jcb.202208088 PMC10148236 37115958

[BIO062632C40] Orsi, A., Razi, M., Dooley, H. C., Robinson, D., Weston, A. E., Collinson, L. M. and Tooze, S. A. (2012). Dynamic and transient interactions of Atg9 with autophagosomes, but not membrane integration, are required for autophagy. *Mol. Biol. Cell* 23, 1860-1873. 10.1091/mbc.e11-09-0746 PMC3350551 22456507

[BIO062632C41] Orth, J. D., Krueger, E. W., Cao, H. and McNiven, M. A. (2002). The large GTPase dynamin regulates actin comet formation and movement in living cells. *Proc. Natl. Acad. Sci. USA* 99, 167-172. 10.1073/pnas.012607899 PMC117533 11782546

[BIO062632C42] Osawa, T., Kotani, T., Kawaoka, T., Hirata, E., Suzuki, K., Nakatogawa, H., Ohsumi, Y. and Noda, N. N. (2019). Atg2 mediates direct lipid transfer between membranes for autophagosome formation. *Nat. Struct. Mol. Biol.* 26, 281-288. 10.1038/s41594-019-0203-4 30911189

[BIO062632C43] Otera, H., Wang, C., Cleland, M. M., Setoguchi, K., Yokota, S., Youle, R. J. and Mihara, K. (2010). Mff is an essential factor for mitochondrial recruitment of Drp1 during mitochondrial fission in mammalian cells. *J. Cell Biol.* 191, 1141-1158. 10.1083/jcb.201007152 PMC3002033 21149567

[BIO062632C44] Puri, C., Vicinanza, M., Ashkenazi, A., Gratian, M. J., Zhang, Q., Bento, C. F., Renna, M., Menzies, F. M. and Rubinsztein, D. C. (2018). The RAB11A-positive compartment is a primary platform for autophagosome assembly mediated by WIPI2 recognition of PI3P-RAB11A. *Dev. Cell* 45, 114-131.e8. 10.1016/j.devcel.2018.03.008 PMC5896254 29634932

[BIO062632C45] Ran, F. A., Hsu, P. D., Wright, J., Agarwala, V., Scott, D. A. and Zhang, F. (2013). Genome engineering using the CRISPR-Cas9 system. *Nat. Protoc.* 8, 2281-2308. 10.1038/nprot.2013.143 PMC3969860 24157548

[BIO062632C46] Ravenhill, B. J., Boyle, K. B., von Muhlinen, N., Ellison, C. J., Masson, G. R., Otten, E. G., Foeglein, A., Williams, R. and Randow, F. (2019). The cargo receptor NDP52 initiates selective autophagy by recruiting the ULK complex to cytosol-invading bacteria. *Mol. Cell* 74, 320-329.e6. 10.1016/j.molcel.2019.01.041 PMC6477152 30853402

[BIO062632C47] Renard, H. F., Simunovic, M., Lemiere, J., Boucrot, E., Garcia-Castillo, M. D., Arumugam, S., Chambon, V., Lamaze, C., Wunder, C., Kenworthy, A. K. et al. (2015). Endophilin-A2 functions in membrane scission in clathrin-independent endocytosis. *Nature* 517, 493-496. 10.1038/nature14064 PMC4342003 25517096

[BIO062632C48] Sawa-Makarska, J., Baumann, V., Coudevylle, N., von Bulow, S., Nogellova, V., Abert, C., Schuschnig, M., Graef, M., Hummer, G. and Martens, S. (2020). Reconstitution of autophagosome nucleation defines Atg9 vesicles as seeds for membrane formation. *Science* 369, eaaz7714. 10.1126/science.aaz7714 PMC7610778 32883836

[BIO062632C49] Schmid, S. L. and Frolov, V. A. (2011). Dynamin: functional design of a membrane fission catalyst. *Annu. Rev. Cell Dev. Biol.* 27, 79-105. 10.1146/annurev-cellbio-100109-104016 21599493

[BIO062632C50] Schulze, R. J., Weller, S. G., Schroeder, B., Krueger, E. W., Chi, S., Casey, C. A. and McNiven, M. A. (2013). Lipid droplet breakdown requires dynamin 2 for vesiculation of autolysosomal tubules in hepatocytes. *J. Cell Biol.* 203, 315-326. 10.1083/jcb.201306140 PMC3812963 24145164

[BIO062632C51] Simpson, F., Hussain, N. K., Qualmann, B., Kelly, R. B., Kay, B. K., McPherson, P. S. and Schmid, S. L. (1999). SH3-domain-containing proteins function at distinct steps in clathrin-coated vesicle formation. *Nat. Cell Biol.* 1, 119-124. 10.1038/10091 10559884

[BIO062632C52] Soreng, K., Munson, M. J., Lamb, C. A., Bjorndal, G. T., Pankiv, S., Carlsson, S. R., Tooze, S. A. and Simonsen, A. (2018). SNX18 regulates ATG9A trafficking from recycling endosomes by recruiting Dynamin-2. *EMBO Rep.* 19, e44837. 10.15252/embr.201744837 PMC5891424 29437695

[BIO062632C53] Stanley, R. E., Ragusa, M. J. and Hurley, J. H. (2014). The beginning of the end: how scaffolds nucleate autophagosome biogenesis. *Trends Cell Biol.* 24, 73-81. 10.1016/j.tcb.2013.07.008 PMC3877172 23999079

[BIO062632C54] Sun, Y., Vashisht, A. A., Tchieu, J., Wohlschlegel, J. A. and Dreier, L. (2012). Voltage-dependent anion channels (VDACs) recruit Parkin to defective mitochondria to promote mitochondrial autophagy. *J. Biol. Chem.* 287, 40652-40660. 10.1074/jbc.M112.419721 PMC3504778 23060438

[BIO062632C55] Takahashi, Y., Coppola, D., Matsushita, N., Cualing, H. D., Sun, M., Sato, Y., Liang, C., Jung, J. U., Cheng, J. Q., Mule, J. J. et al. (2007). Bif-1 interacts with Beclin 1 through UVRAG and regulates autophagy and tumorigenesis. *Nat. Cell Biol.* 9, 1142-1151. 10.1038/ncb1634 PMC2254521 17891140

[BIO062632C56] Takahashi, Y., Tsotakos, N., Liu, Y., Young, M. M., Serfass, J., Tang, Z., Abraham, T. and Wang, H. G. (2016). The Bif-1-Dynamin 2 membrane fission machinery regulates Atg9-containing vesicle generation at the Rab11-positive reservoirs. *Oncotarget* 7, 20855-20868. 10.18632/oncotarget.8028 PMC4991497 26980706

[BIO062632C57] Thompson, H. M., Skop, A. R., Euteneuer, U., Meyer, B. J. and McNiven, M. A. (2002). The large GTPase dynamin associates with the spindle midzone and is required for cytokinesis. *Curr. Biol.* 12, 2111-2117. 10.1016/S0960-9822(02)01390-8 PMC3690653 12498685

[BIO062632C58] Turco, E., Witt, M., Abert, C., Bock-Bierbaum, T., Su, M. Y., Trapannone, R., Sztacho, M., Danieli, A., Shi, X., Zaffagnini, G. et al. (2019). FIP200 claw domain binding to p62 promotes autophagosome formation at ubiquitin condensates. *Mol. Cell* 74, 330-346.e11. 10.1016/j.molcel.2019.01.035 PMC6477179 30853400

[BIO062632C59] Valverde, D. P., Yu, S., Boggavarapu, V., Kumar, N., Lees, J. A., Walz, T., Reinisch, K. M. and Melia, T. J. (2019). ATG2 transports lipids to promote autophagosome biogenesis. *J. Cell Biol.* 218, 1787-1798. 10.1083/jcb.201811139 PMC6548141 30952800

[BIO062632C60] van der Bliek, A. M., Redelmeier, T. E., Damke, H., Tisdale, E. J., Meyerowitz, E. M. and Schmid, S. L. (1993). Mutations in human dynamin block an intermediate stage in coated vesicle formation. *J. Cell Biol.* 122, 553-563. 10.1083/jcb.122.3.553 PMC2119674 8101525

[BIO062632C61] Yamamoto, H., Kakuta, S., Watanabe, T. M., Kitamura, A., Sekito, T., Kondo-Kakuta, C., Ichikawa, R., Kinjo, M. and Ohsumi, Y. (2012). Atg9 vesicles are an important membrane source during early steps of autophagosome formation. *J. Cell Biol.* 198, 219-233. 10.1083/jcb.201202061 PMC3410421 22826123

[BIO062632C62] Yamamoto, H., Zhang, S. and Mizushima, N. (2023). Autophagy genes in biology and disease. *Nat. Rev. Genet.* 24, 382-400. 10.1038/s41576-022-00562-w PMC9838376 36635405

[BIO062632C63] Yu, L., Chen, Y. and Tooze, S. A. (2017). Autophagy pathway: cellular and molecular mechanisms. *Autophagy* 14, 207-215. 10.1080/15548627.2017.1378838 PMC5902171 28933638

