# Supplementary material for: Dynamin-2 promotes Atg9A retrieval from phagophores during autophagy
Source: Biol Open. 2026 Oct 16;15(10):bio062632. doi: 10.1242/bio.062632 (PMC13714872; doi:10.1242/bio.062632)
Supplement: Supplementary file 1 [file biolopen-15-062632-s1.pdf]

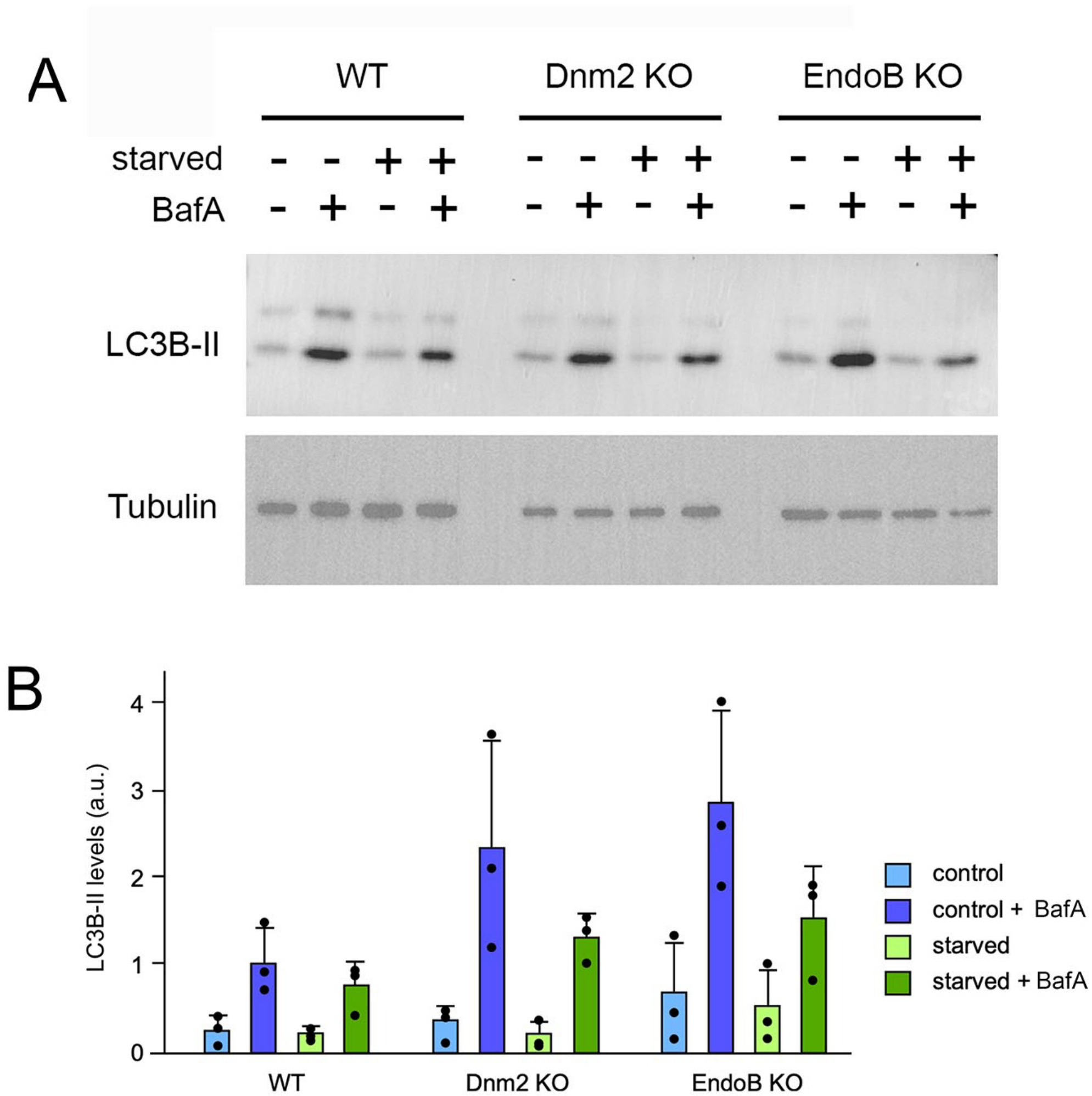

Fig. S1. Colocalization of Dnm2 and EndoB1 shown with scatterplots.

(A) Scatterplots of immunofluorescence images of cells treated for 2 h with or without rapamycin to induce autophagy. **(B)** Fraction of spots in the colocalizing quadrants of the scatterplots, shown before and after 2 or 4 h of treatment with rapamycin or CCCP. Average and SD are shown for three biological replicates, each determined with five technical repeats. Statistical analysis used a one-way ANOVA with Dunnett's post hoc test across biological replicates. **(C,D)** As in panels A and B, but with transiently expressed GFP- and RFP-tagged proteins. **(E)** Western blots showing the derivation of Dnm2, EndoB1, and DKO cells (clone A4). Dnm2 and EndoB1 KO cells were generated by CRISPR/Cas9 in MEFs. DKO cells were generated by additional knockout of EndoB1 in Dnm2 KO cells, and clone A4 was chosen for further studies. **(F)** PLA signals for Dnm2 and EndoB1 interactions were absent from cells with loss of one or both of these proteins. Scale bar is 10 µm. **(G)** As a negative control for PLA, cells were incubated with Fis1 and Hsp60 antibodies, which detect proteins on the outer or inner surface of mitochondria and are therefore too far apart to generate PLA signals. As a positive control, cells were incubated with antibodies for Drp1 and Mff, which detect well-characterized binding partners. Scale bar is 10 µm.

Biology Open • Supplementary information

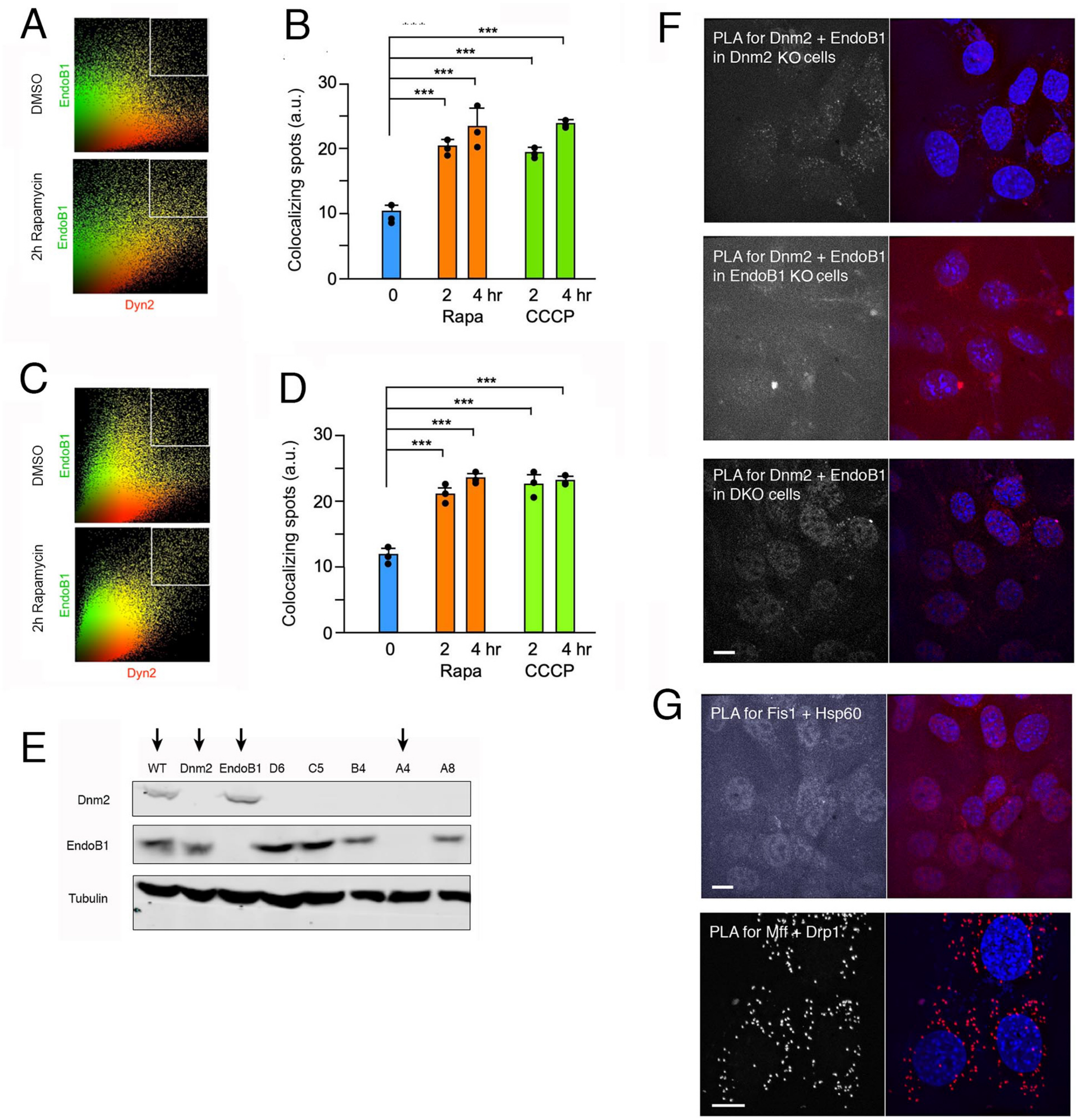

Fig. S2. Effects of EndoB KO on mitophagy in MEFs and of Dnm2 and EndoB KO on starvation-induced LC3B lipidation.

(A) Western blots showing the effects of starvation-induced macroautophagy and Bafilomycin A treatments on LC3B lipidation in wildtype, Dnm2 and EndoB KO HeLa cells. **(B)** Average intensities of LC3B-II bands in four independent experiments, relative to tubulin levels, expressed in arbitrary units (a.u.). Average and SD are shown for three biological replicates. Statistical analysis using one-way ANOVA with Tukey's post hoc comparisons did not show a significant difference.

Biology Open • Supplementary information

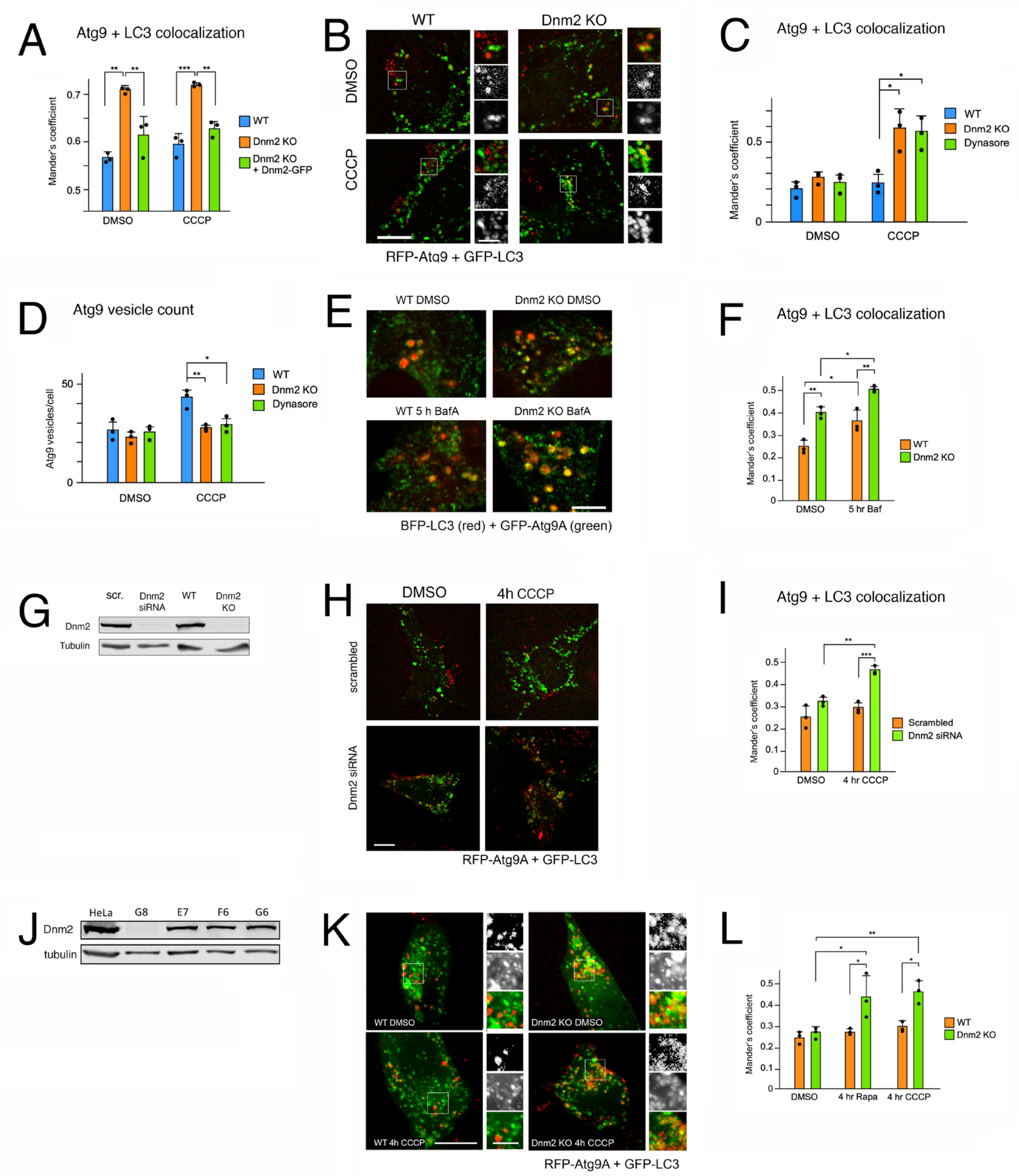
A
Atg9 + LC3 colocalization
Mander's coefficient
0.5
0.6
0.7
DMSO
CCCP
WT
Dnm2 KO
Dnm2 KO + Dnm2-GFP
B
WT
Dnm2 KO
DMSO
CCCP
RFP-Atg9 + GFP-LC3
C
Atg9 + LC3 colocalization
Mander's coefficient
0
0.5
DMSO
CCCP
WT
Dnm2 KO
Dynasore
D
Atg9 vesicle count
Atg9 vesicles/cell
0
50
DMSO
CCCP
WT
Dnm2 KO
Dynasore
E
WT DMSO
Dnm2 KO DMSO
WT 5 h BafA
Dnm2 KO BafA
BFP-LC3 (red) + GFP-Atg9A (green)
F
Atg9 + LC3 colocalization
Mander's coefficient
DMSO
5 hr Baf
WT
Dnm2 KO
G
scr.
Dnm2 siRNA
WT
Dnm2 KO
Dnm2
Tubulin
H
DMSO
4h CCCP
scrambled
Dnm2 siRNA
RFP-Atg9A + GFP-LC3
I
Atg9 + LC3 colocalization
Mander's coefficient
DMSO
4 hr CCCP
Scrambled
Dnm2 siRNA
J
HeLa
G8
E7
F6
G6
Dnm2
tubulin
K
WT DMSO
Dnm2 KO DMSO
WT 4h CCCP
Dnm2 KO 4h CCCP
RFP-Atg9A + GFP-LC3
L
Mander's coefficient
DMSO
4 hr Rapa
4 hr CCCP
WT
Dnm2 KO

Fig. S3. Atg9A localization in wt and Dnm2 siRNA MEF cells and in Dnm2 KO HeLa cells. (A) Histogram of rescue experiment showing a decrease in Mander's coefficient for colocalization of Atg9A-myc and HA-LC3 for Dnm2 KO MEFs upon transfection with Dnm2-EGFP. The results show similar decreases for treatments with DMSO or CCCP. Average and SD are shown for three biological replicates, with five technical replicates per biological replicate. Statistical analysis used one-way ANOVA with Tukey's post hoc comparisons. **(B)** Live-cell fluorescence images show that RFP-Atg9A colocalizes with EGFP-LC3 in Dnm2 KO MEFs treated with CCCP, but not in wild-type MEFs. Scale bar is 10 μm for whole cells and 3 μm for enlarged portions. **(C)** Histogram showing Mander's coefficient for colocalization of RFP-ATG9A and GFP-LC3 in Dnm2 KO MEFs and dynasore-treated WT MEFs after incubation with rapamycin or CCCP. Average and SD are shown for three biological replicates, with five technical replicates per biological replicate. Statistical analysis used one-way ANOVA with Tukey's post hoc comparisons. **(D)** The number of Atg9A vesicles (RFP spots without GFP label) decreases when Dnm2 KO MEFs and dynasore-treated WT MEFs are incubated with rapamycin or CCCP. Statistics as in panel B. **(E, F)** Effects of Dnm2 on Atg9A retrieval in live cells. Images of BFP- LC3 (shown in red) and GFP-Atg9A in live WT and Dnm2 KO cells, untreated or after 5 hr with Bafilomycin. Scale bar is 5 μm. Histograms showing increased colocalization of BFP-LC3 and GFP-Atg9A in Dnm2 KO cells with or without Bafilomycin treatments. Average and SD are shown for three biological replicates, each with five technical replicates. Statistical analysis used one-way ANOVA with Tukey's post hoc comparisons. Scale bar is 10 μm. **(G)** Western blot showing Dnm2 depletion after transfection of WT cells with siRNA oligonucleotides. **(H)** RFP-Atg9A colocalizes with EGFP-LC3 after 4h treatment with CCCP in Dnm2 siRNA cells, but not in mock-transfected cells. Scale bar is 10 μm. **(I)** Histogram showing increased colocalization of RFP-ATG9A and GFP-LC3 in CCCP-treated Dnm2 siRNA cells. Average and SD are shown for three biological replicates, each with five technical replicates. Statistical analysis used one-way ANOVA with Tukey's post hoc comparisons. **(J)** Western blot showing depletion of Dnm2 in a CRISPR/Cas9-generated HeLa cell clones. Clone G8 was chosen for further analysis. **(K)** RFP-Atg9A colocalizes with EGFP-LC3 in Dnm2 KO HeLa cells treated with CCCP, but not in wild type cells. Scale bar is 10 μm for whole cells and 3 μm for enlarged portions. **(L)** Histogram showing increased colocalization of RFP-ATG9A and GFP-LC3 in Dnm2 KO HeLa cells after incubation with rapamycin or CCCP. Average and SD are shown for three biological replicates, with five technical replicates per biological replicate. Statistical analysis used one-way ANOVA with Tukey's post hoc comparisons.

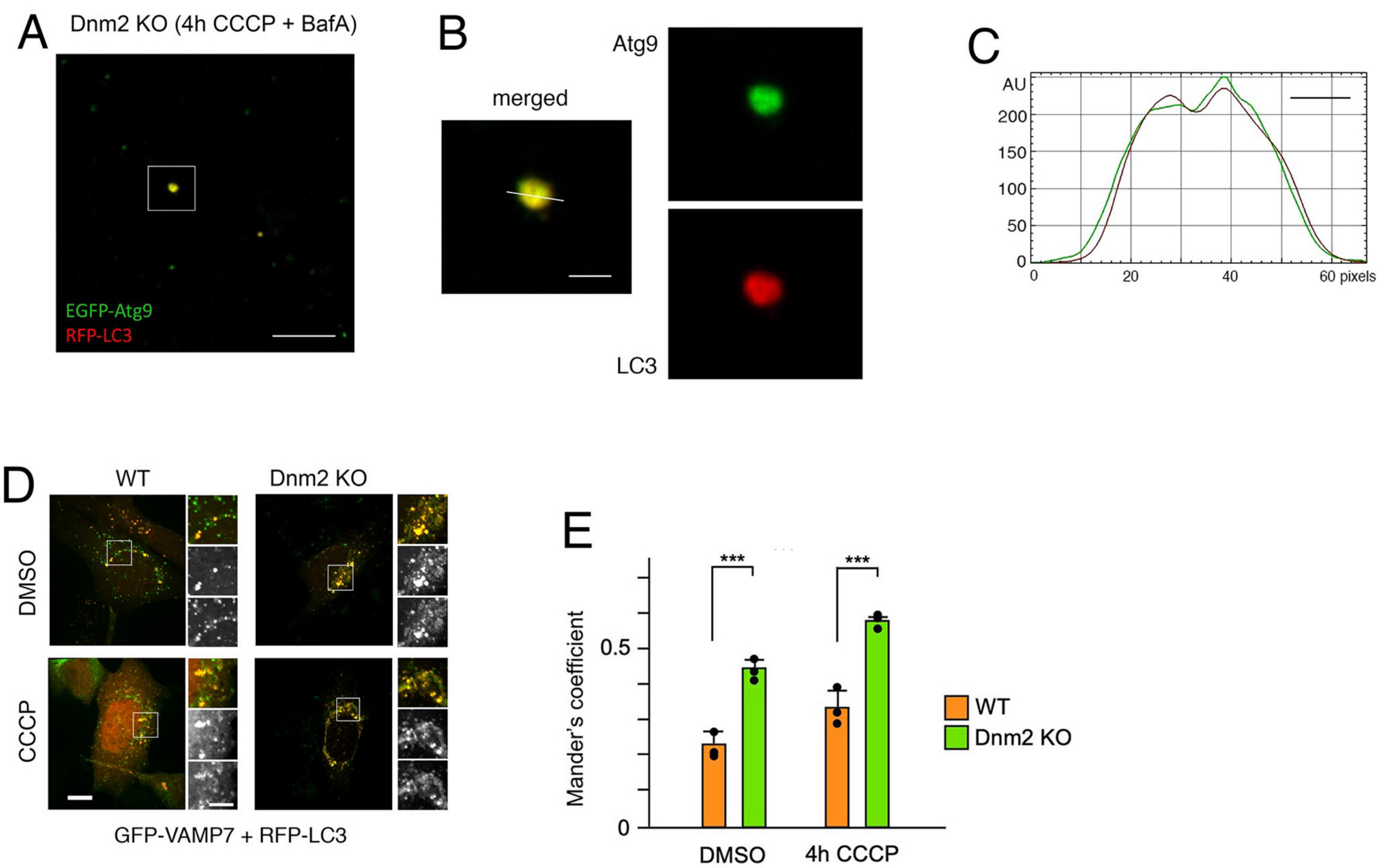

Fig. S4. Colocalization of GFP-Atg9A and RFP-LC3 in autophagosome fractions and of GFP-VAMP7 and RFP-LC3 in live cells.

(A) Image of autophagosome-containing fraction from Dnm2 KO cells transfected with EGFP-Atg9A and RFP-LC3, followed by 4hr treatments with CCCP and Bafilomycin to induce mitophagy and to accumulate autophagosomes. Autophagosomes were enriched by differential centrifugation. Scale bar is 10 μm. **(B, C)** Enlargements show an individual spot with the line used to plot intensities. Scale bar is 2 μm. The x-axis in the fluorescence intensity plot shows numbers of pixels (24 pixel/μm) and the bar shows 500 nm. **(D, E)** Colocalization of GFP-VAMP7 with RFP-LC3 is higher in Dnm2 KO MEFs than wildtype cells upon treatment with CCCP. Scale bar is 10 μm. Average and SD are shown for three biological replicates, with five technical replicates per biological replicate. Statistical analysis used one-way ANOVA with Tukey's post hoc comparisons.

Biology Open • Supplementary information

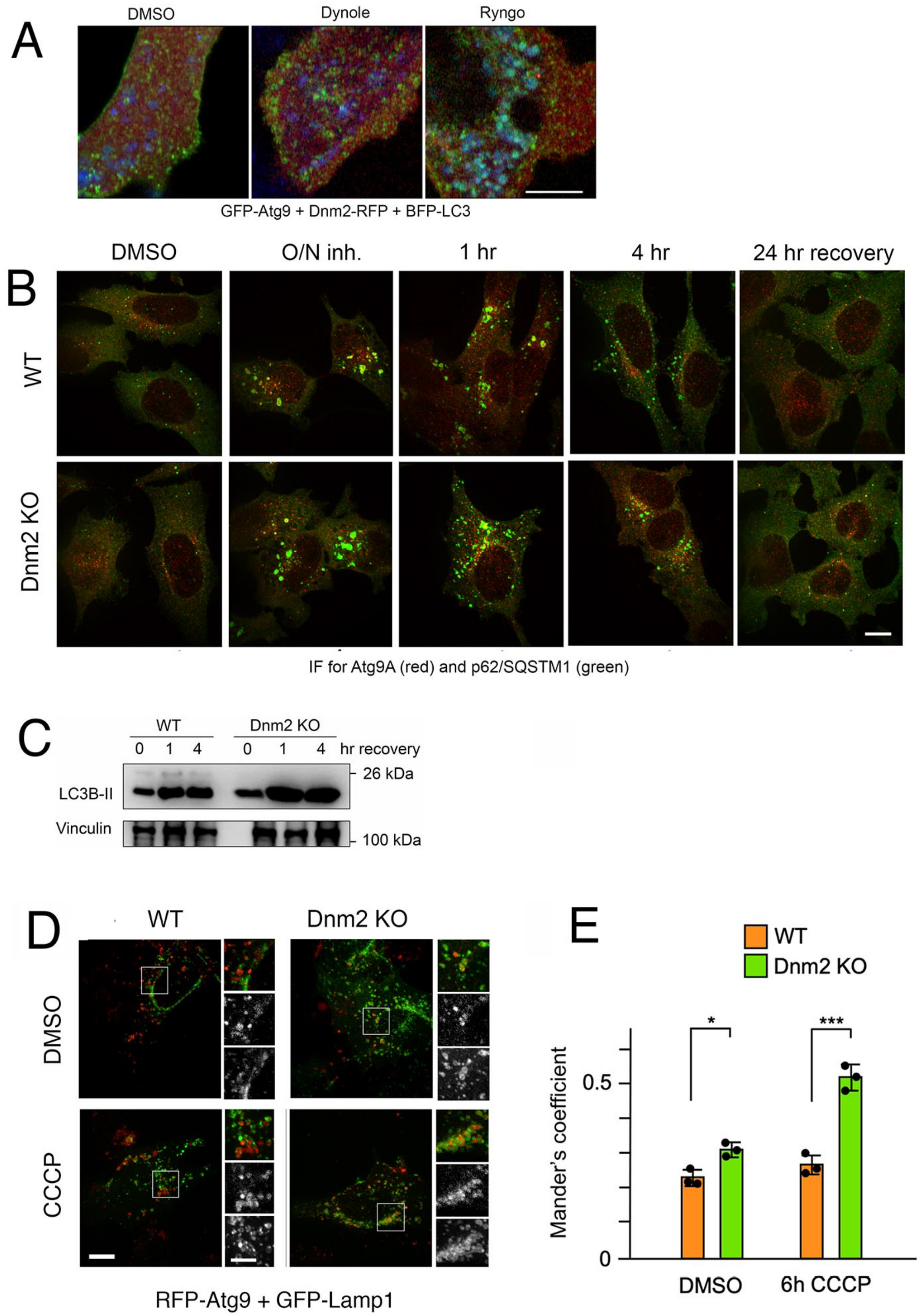
A
DMSO
Dynole
Ryngo
GFP-Atg9 + Dnm2-RFP + BFP-LC3
B
DMSO
O/N inh.
1 hr
4 hr
24 hr recovery
WT
Dnm2 KO
IF for Atg9A (red) and p62/SQSTM1 (green)
C
WT
Dnm2 KO
0 1 4 0 1 4 hr recovery
26 kDa
LC3B-II
Vinculin
100 kDa
D
WT
Dnm2 KO
DMSO
CCCP
RFP-Atg9 + GFP-Lamp1
E
WT
Dnm2 KO
*
***
Mander's coefficient
0.5
0
DMSO
6h CCCP

Fig. S5. Representative images of Dnm2 colocalization with Atg9A and LC3, from colocalization of Atg9A with p62/SQSTM1 and with LAMP1.

(**A**) Representative images of whole cells taken from time-lapse series of Dnm2-RFP, GFP-Atg9A, and BFP-LC3 spots in untreated cells and cells treated with Dynole 34-2 or Ryngo 1-23. Scale bar is 10 μm. **(B)** Representative images showing delayed dissociation of Atg9A and p62 in Dnm2 KO cells. A buildup of Atg9A and p62 was induced for 16h with the Vps34 inhibitor Vps34-IN1. This was followed by inhibitor washout for the indicated durations. Association and dissociation of Atg9A (red) and p62 (green) were monitored by immunofluorescence microscopy of endogenous proteins in WT and Dnm2 KO HeLa cells. Scale bar is 10 μm. (**C)** Western blot showing that the recovery of LC3B-II after removal of VPS34inh is unimpeded in Dnm2 KO cells. Vinculin is a loading control. (**D, E**) Increased colocalization between RFP-Atg9 and GFP-LAMP1 after CCCP treatment. Cells were incubated with pepstatin and E64d to prevent Atg9A digestion in lysosomes. Scale bar is 10 μm for whole cells and 5 μm for enlarged portions. Average and SD are shown for three biological replicates, with five technical replicates per biological replicate. Statistical analysis used one-way ANOVA with Tukey's post hoc comparisons.

Figure 2C

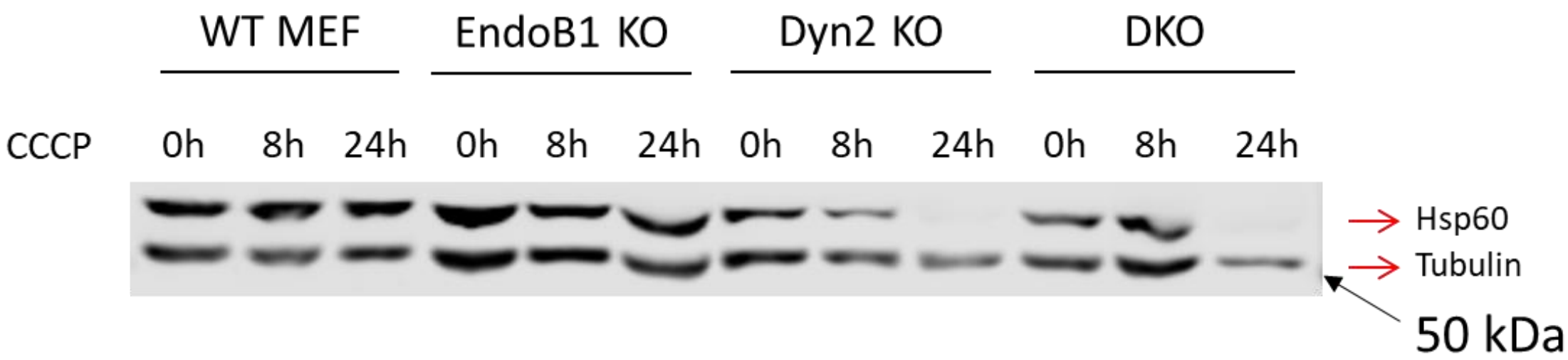

Figure 2E

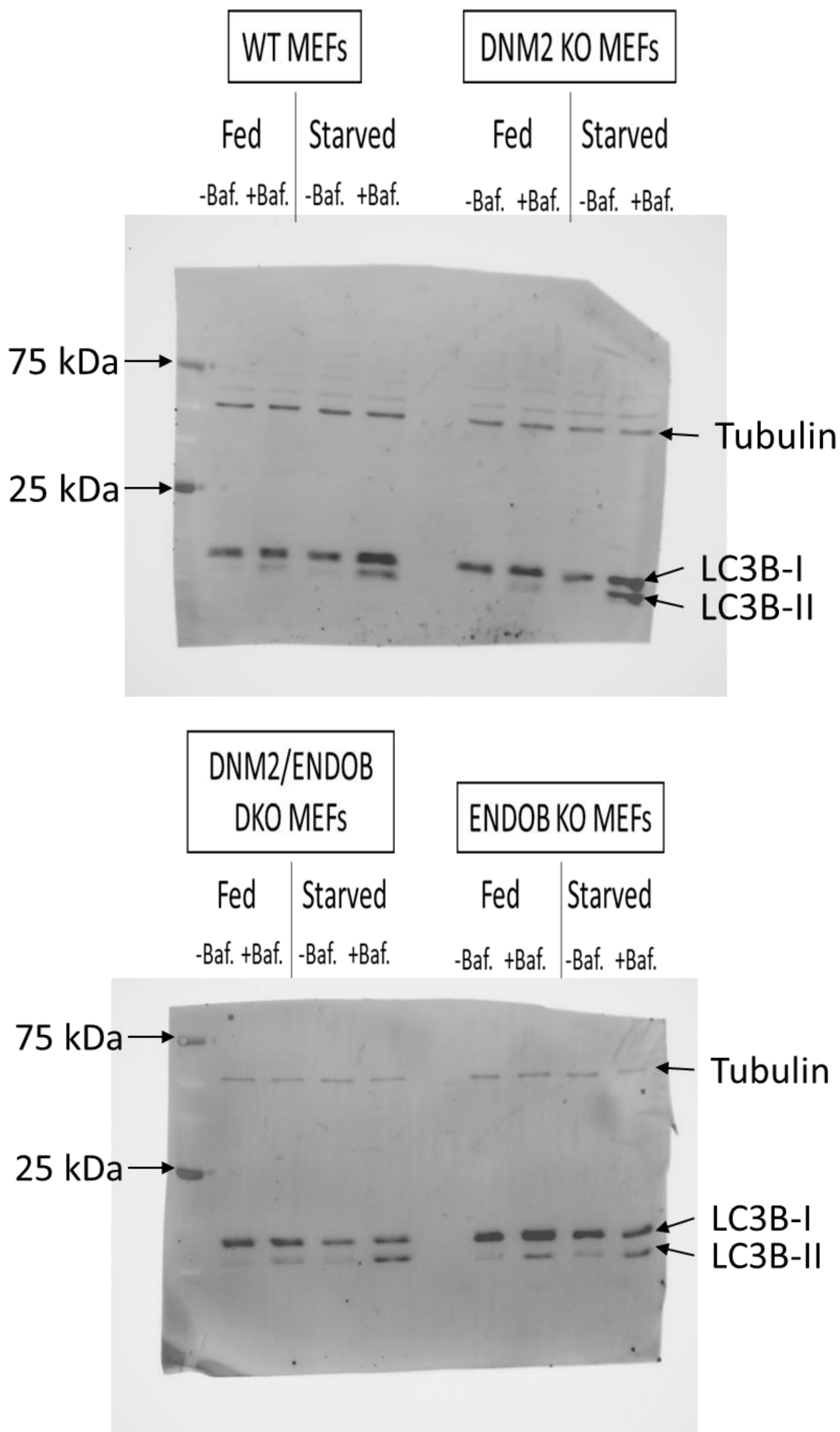

Figure 4C

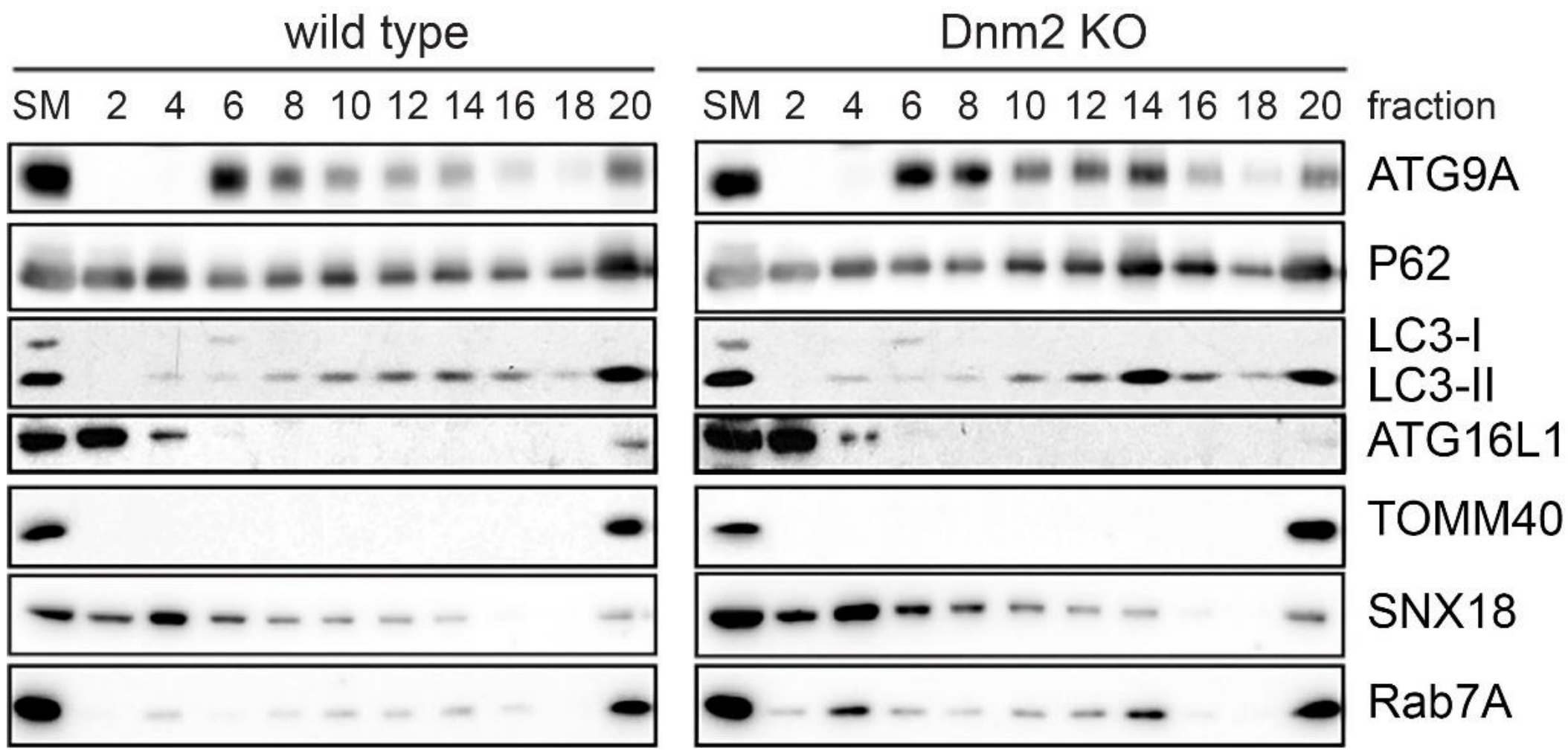

Figure 5F

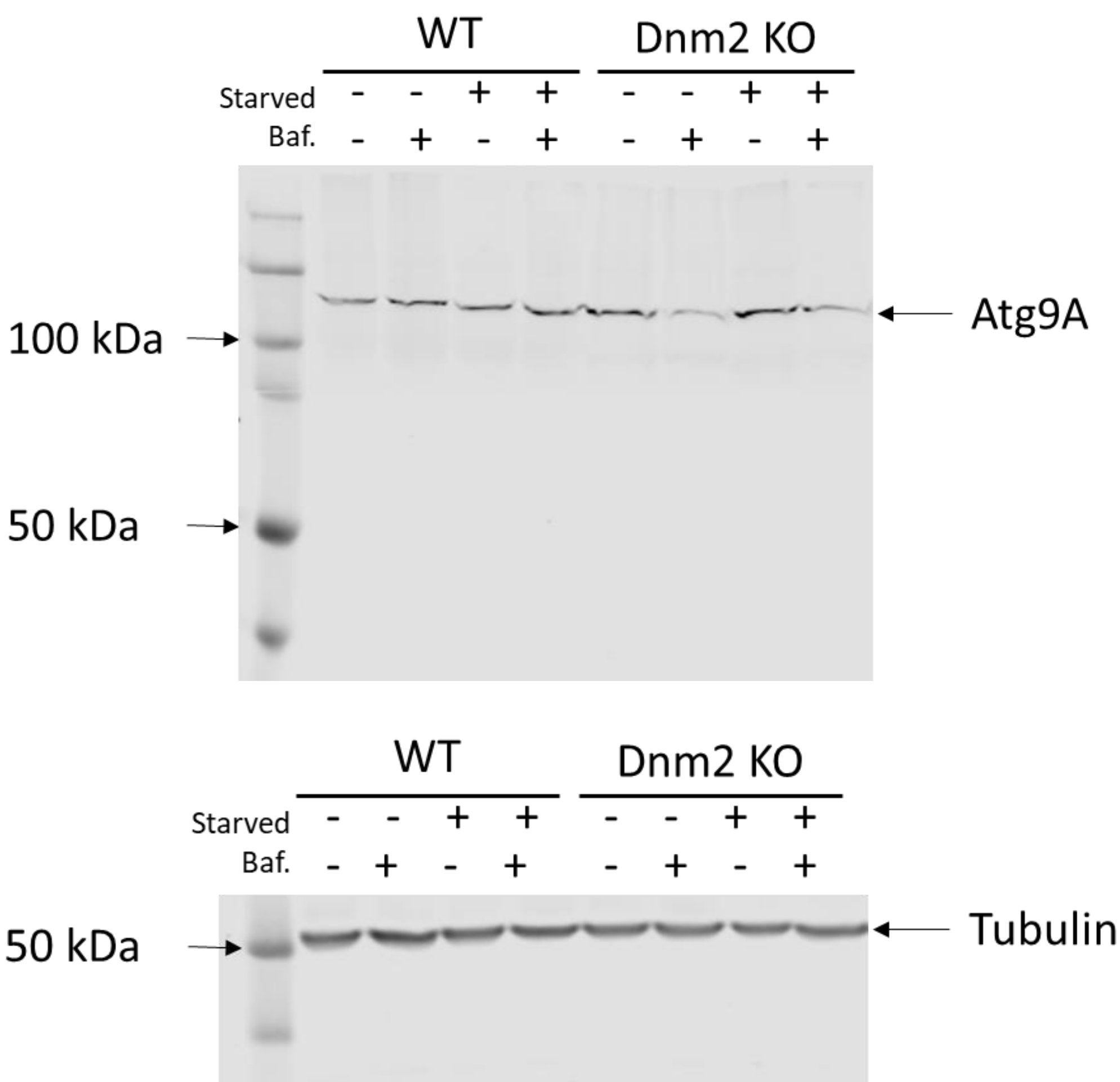

Biology Open • Supplementary information

Suppl. Figure 1E

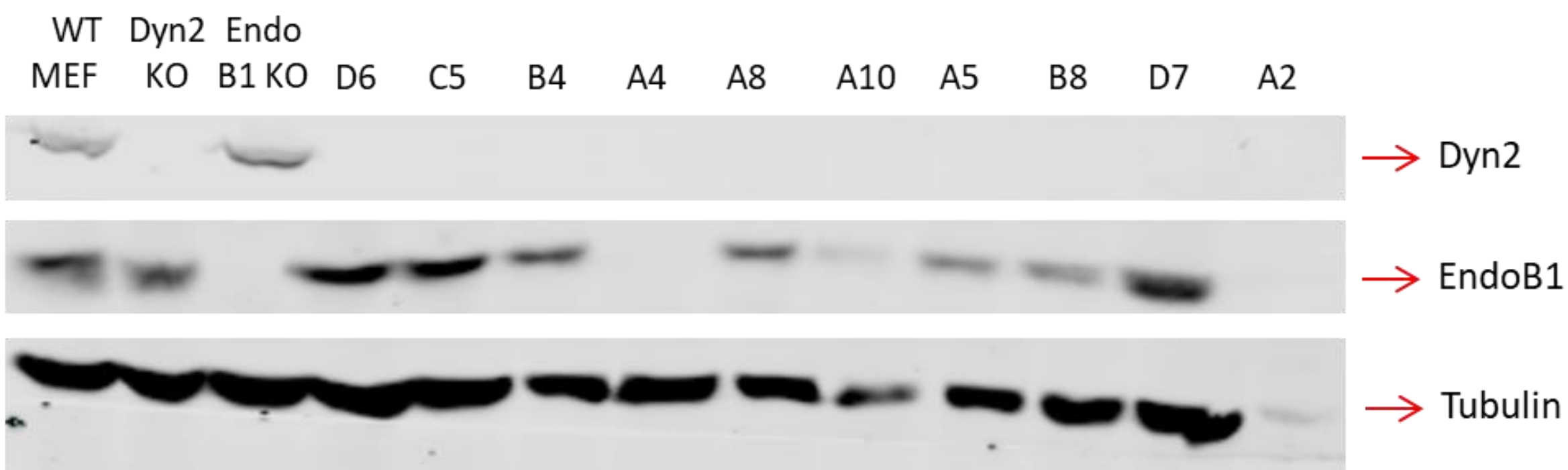

Suppl. Figure 2E

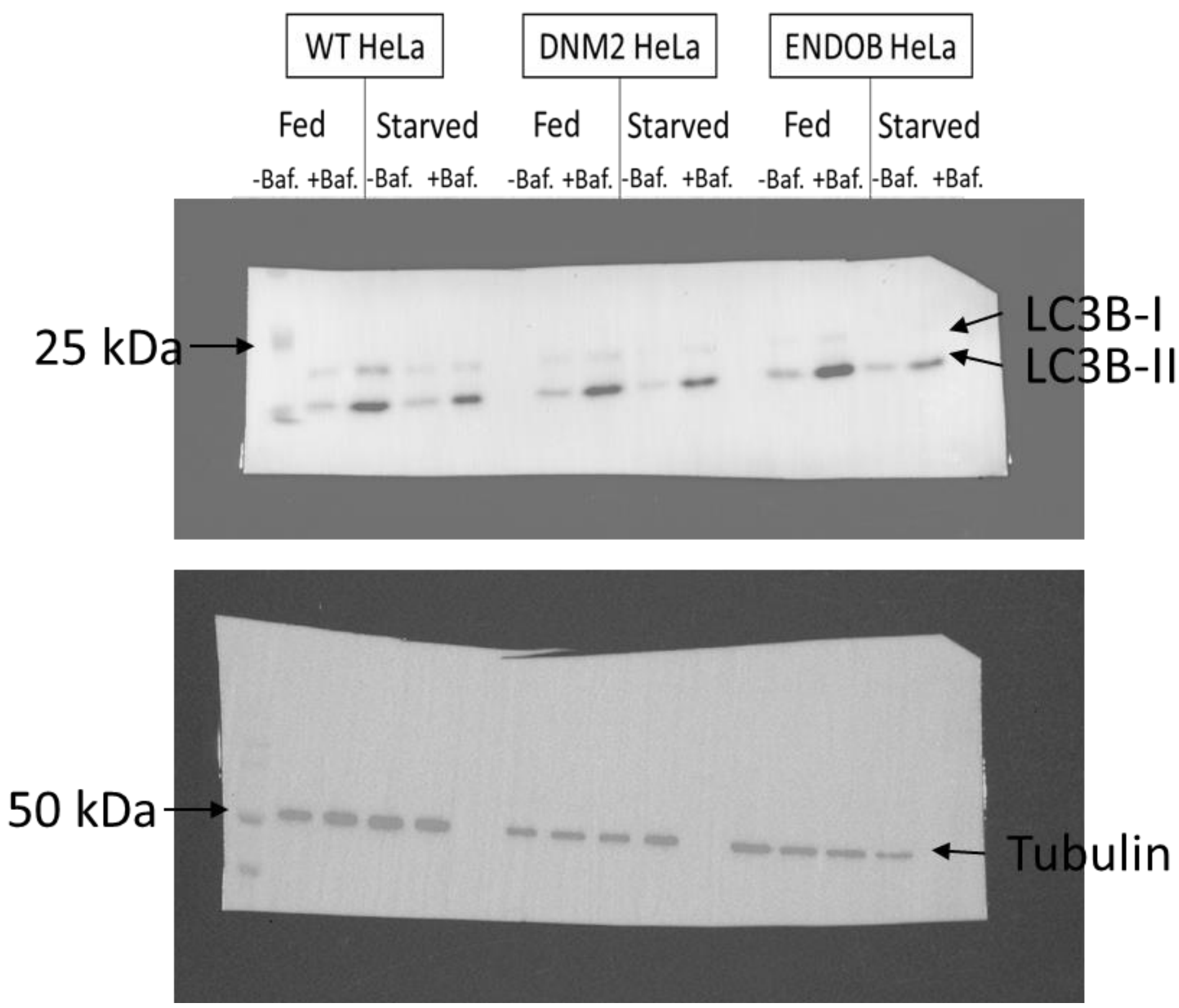

Biology Open • Supplementary information

Suppl. Figure 3F

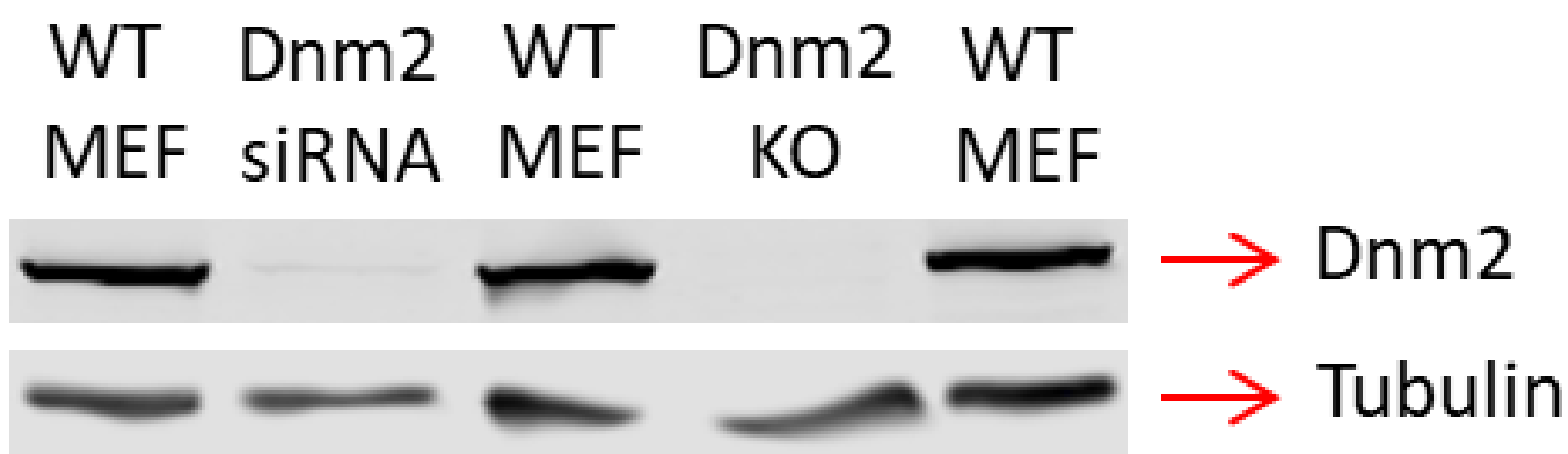

Suppl. Figure 3I

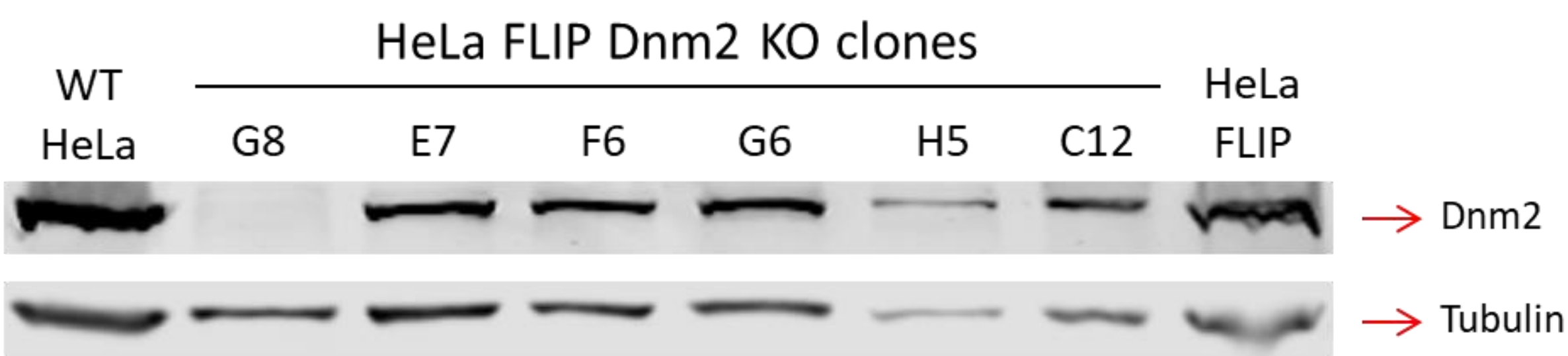

Fig. S6. Western blot transparency.

Table S1. Source and catalog numbers of antibodies used in this study.

Immunogen	Host	Company	Cat nr
ATG9A	Rabbit	Proteintech	26276-1-AP
Cytochrome c	Mouse	BD Biosciences	556432
Dnm2	Mouse	Proteintech	68209-1-IG
Dnm2	Goat	Santa Cruz Biotechnology	SC-6400
Drp1 (DLP1)	Mouse	BD Biosciences	611113
Endophilin A1	Rabbit	Abcam	Ab169762
Endophilin B1	Mouse	Imgenex	IMG-265
Hsp60	Mouse	Abcam	AB59457
LC3B	Rabbit	Sigma Aldrich	L7543-200UL
RAB7A	Rabbit	Proteintech	55469-1-AP
SNX18	Rabbit	GeneTex	GTX119426
SQSTM1 (p62)	Mouse	Abcam	AB91526
Tim23	Mouse	BD Biosciences	611223
Tom20	Rabbit	Proteintech	11802-1-AP
Tubulin	Mouse	Sigma Aldrich	T 6199
Vinculin	Mouse	Sigma Aldrich	V9131-100UG
VDAC1/Porin	Mouse	Abcam	AB34726

Biology Open • Supplementary information
