# Supplementary material for: Dynamin-2 promotes Atg9A retrieval from phagophores during autophagy
Source: Biol Open. 2026 Oct 16;15(10):bio062632. doi: 10.1242/bio.062632 (PMC13714872; doi:10.1242/bio.062632)
Supplement: Supplementary file 2 [file biolopen-15-062632_review_history.pdf]

Dynamin-2 promotes Atg9A retrieval from phagophores during autophagy

Andrew Caliri, Alejandro Martorell Riera, Akash Saha, Panagiota Kolitsida, Cinta Iriondo Martinez, Samuel Itskanov, Janos Steffen, Carla M. Koehler and Alex Van der Bliek
10.1242/bio.062632

Editor: Catherine L. Jackson

Review timeline

Original submission:	15 April 2026
Editorial decision:	24 April 2026
First revision received:	24 August 2026
Accepted:	27 August 2026

Original submission

First decision letter

MS ID#: bio.062632

MS Title: Dynamin-2 promotes Atg9A retrieval from phagophores during autophagy

Authors: Alex Van der Bliek; Andrew Caliri; Alejandro Martorell Riera; Akash Saha; Panagiota Kolitsida; Cinta Iriondo Martinez; Samuel Itskanov; Janos Steffen; Carla M. Koehler

I have now reached a decision on the above manuscript.

The reviewer reports are shown at the bottom of this email.

As you will see, the reviewers gave favourable reports, but raised some critical points that will require amendments to your manuscript. I hope that you will be able to carry these out, because we would like to be able to accept your paper.

A major criterion for publication in Biology Open is that the data presented support the conclusions drawn in the paper. For your manuscript, either further experiments can be carried out to address the points raised, or changes made to the text such that the conclusions are fully supported by the data presented.

At this stage, we also ask you to ensure your manuscript complies with our formatting guidelines – please see our manuscript preparation guidelines for details. Provided you are able to fully address the referees' comments, we are positive about publication of your paper (we accept over 95% of revision submissions) and therefore hope you won't mind any extra work involved in reformatting your manuscript at this point.

Please upload both a 'clean' version of your Word file, along with a highlighted version clearly showing where you have made changes in the revised manuscript. Please avoid using 'Track changes' in Word files as these are lost in PDF conversion.

I should be grateful if you would also provide a point-by-point response detailing how you have dealt with the points raised by the reviewers in the 'Response to Reviewers' box. Please attend to

© 2026. Published by The Company of Biologists under the terms of the Creative Commons Attribution License (https://creativecommons.org/licenses/by/4.0/).

all of the reviewers' comments. If you do not agree with any of their criticisms or suggestions please explain clearly why this is so.

Reviewer 1

Comments for the author

This manuscript presents an interesting and potentially important model in which Dnm2 promotes Atg9A retrieval from phagophores during autophagy. The study uses multiple complementary approaches and addresses a significant mechanistic question in the autophagy field, making it potentially suitable for publication. The overall conclusions are intriguing and potentially suitable for publication; however, three major issues should be addressed to strengthen the mechanistic conclusions, along with several minor revisions related to clarity, consistency, and figure presentation.

Major comments:

1. Missing genetic rescue is a major gap: A significant weakness of the study is the absence of genetic rescue experiments. The authors rely on knockout cells and pharmacological perturbations to support a role for Dnm2 in Atg9A retrieval, but do not demonstrate restoration of the phenotype by re-expression of Dnm2. This would be important to confirm the specificity of the phenotype. At a minimum, the central model would be significantly strengthened by the rescue of Atg9A retrieval defects with WT Dnm2.

2. The direct membrane scission mechanism remains inferred rather than demonstrated. While the data support a role for Dnm2 in Atg9A retrieval, the stronger claim that Dnm2 directly mediates membrane scission at phagophores remains somewhat inferential. The authors should either provide stronger mechanistic evidence supporting direct scission or temper the wording throughout the manuscript where Dnm2 is described as the scission factor.

3. The pulse-chase approach is elegant and potentially informative, but several controls are needed to support the interpretation that delayed Atg9A-p62 dissociation reflects defective Atg9A retrieval specifically.

* Verification that Vps34 inhibition effectively blocks autophagic flux.

* Demonstration that LC3 lipidation recovers after Vps34-IN1 washout

* Evidence that delayed Atg9A-p62 dissociation in Dnm2 KO cells does not simply reflect delayed recovery of autophagy, rather than a specific retrieval defect.

Minor comments:

1. Figure 1:

A. In the PLA experiment (1E, F), although the text states all autophagy-inducing conditions significantly increased PLA, statistics appear to be shown only for AntA. Please provide a statistical analysis for all conditions discussed.

B. Related to results in Fig 1: Page 4, Lines 7-10: The sentence beginning "The increase in PLA spots…" appears redundant and could be removed.

C. Figure1 F-H: The presentation and labeling of the PLA data should be clarified and standardized. In Fig. 1F and G, y-axis terminology is inconsistent ("spots/cell" vs "PLA spots/cell"), and the legend alternates between "dots" and "spots"; consistent nomenclature should be used throughout. Panel G also refers to "relative numbers," which should be explicitly defined. In addition, the substantially different y-axis ranges between panels (0-50 vs 0-10) should be justified or harmonized to facilitate comparison. Fig. 1H, treatment conditions should be labeled directly on the figure, representative

© 2026. Published by The Company of Biologists under the terms of the Creative Commons Attribution License (https://creativecommons.org/licenses/by/4.0/).

images for the untreated control should be included, and there was a decrease in the fraction of PLA spots associated with LAMP1. Is this a significant decrease?

2. Figures 2/3:

A. Figure 2B: I recommend labeling TOM20 RED and Hsp60 green directly on images. This would improve readability and make the images easier to interpret without requiring the reader to repeatedly refer back to the legend.

B. Figure 2C: Please address the apparent discrepancy between microscopy (complete signal loss) and Western blot (strong remaining WT signal at 24h) and indicate whether degradation between 0h and 24h is significant.

C. Use consistent labeling ("0 h" vs "untreated") across panels. (e.g., Fig. 2C vs 2D)

D. Figure 2E: WT and Dnm2 KO show additional bands on the tubulin blot that are not present in the EndoB KO or the DKO. Why is there an extra band?

E. Please use consistent nomenclature for Bafilomycin A throughout the manuscript, Fig. S2A: "BafA", other figures "Baf".

F. Page 5, line 8: Replace mutations in Dnm2 with loss of Dnm2 or Dnm2 knockout.

G. Page 5, lines 11-15: The interpretation that Fig. 3B demonstrates "more red spots" may overstate what is shown; panel B appears to support increased autophagosomes rather than specifically autolysosomes.

3. Figure presentation/consistency:

A. Figure Presentation: Throughout the manuscript, fluorophore identities should be indicated directly on the figures rather than only in the legends. In several panels (for example, Fig. 4A), the reader must infer which signal corresponds to each marker. I recommend labeling fluorophores within the figures using color-coded text matching the channels (e.g. Atg9A in green, LC3B in red), which would substantially improve readability and allow readers to interpret images without repeatedly referring back to the legends. Figure 4D provides a good example of this approach and could serve as a model for the rest of the manuscript.

B. Supplement Fig. S3: Standardize "untreated" vs "DMSO" labeling and check for similar inconsistencies throughout.

C. Figure 4C: The blot is cropped from the right. Please provide a full, uncropped blot with complete protein labels.

D. Please provide representative images for the Dynole 34-2 and Ryngo 1-23 experiments, potentially as supplemental data.

E. Figure 5C would benefit from representative images accompanying quantification.

4. In the Discussion, the authors state that five lines of evidence support the model that Dnm2 is essential for Atg9A retrieval. Please cite the corresponding figures for each line of evidence explicitly.

5. Materials and methods: Rabbit anti-dynamin 1 is listed in the method section, although it was not used in this study. I also recommend including the catalogue numbers of each antibody used for reproducibility.

Reviewer 2

Comments for the author

© 2026. Published by The Company of Biologists under the terms of the Creative Commons Attribution License (https://creativecommons.org/licenses/by/4.0/).

This manuscript proposes that Dynamin-2 (Dnm2) and Endophilin-B1 (EndoB1) mediate retrieval of Atg9A from phagophores via membrane fission. The biological question is well-motivated. Atg9A is known to be absent from mature autophagosomes (Orsi et al., 2012) but the retrieval mechanism is unknown. The study's multi-method approach (PLA, live-cell imaging, subcellular fractionation, and pharmacological modulation) is a clear strength, and the convergence of these independent datasets is compelling. However, several key issues remain that need to be addressed.

Specific comments

1. Figure 5F-G shows that Atg9A levels decrease in Dnm2 KO cells during CCCP-induced mitophagy and are restored by Bafilomycin A1, indicating that Atg9A undergoes lysosomal degradation in the absence of Dnm2. However, Figures 2-3 suggest that mitophagy and bulk autophagic flux are maintained or even enhanced under these conditions. Given that Atg9A is implicated in supplying membrane for autophagosome formation, sustained depletion would be expected to limit autophagosome biogenesis over time. The manuscript does not address how autophagic activity is maintained despite this apparent loss. Possible explanations include compensatory upregulation of Atg9A synthesis, increased efficiency of Atg9A recycling, or the use of alternative membrane sources. Clarifying whether Atg9A levels recover over time or whether autophagy remains sustained during prolonged stress would help resolve this issue.

2. The authors note that anti-Atg9A immunofluorescence shows lower specificity in mouse cells compared to human cells. However, key colocalization analyses in Figures 4A-B rely on this antibody in MEFs without inclusion of an Atg9A knockout negative control. As a result, the specificity of the staining and therefore the interpretation of Mander's coefficients cannot be fully assessed. Inclusion of Atg9A KO MEFs as a negative control would be important to validate the signal and support the colocalization analysis.

3. The key fractionation experiment (Fig. 4C) relies on Bafilomycin pretreatment to accumulate autophagosomes. However, Bafilomycin elevates lysosomal pH and induces organelle swelling, which can alter buoyant density independently of changes in protein composition. Therefore, the enrichment of Atg9A, LC3-II, and p62 in intermediate fraction #14 in Dnm2 KO cells may reflect Bafilomycin-induced density shifts rather than altered autophagosomal content. Inclusion of a WT + Bafilomycin control (without Rapamycin) would help distinguish these possibilities.

4. The central mechanistic evidence (Fig. 5A) is based on overexpressed Dnm2-RFP, GFP-Atg9A, and BFP-LC3. Given that Dynamin-2 overexpression can alter membrane morphology (including tubulation), it is difficult to exclude the possibility that the observed events reflect overexpression artefacts rather than physiological behavior. The extrapolation from low-frequency events (1 in 3 videos in a single focal plane) to ~25 events per cell per minute relies on assumptions about spatial and temporal sampling that are not explicitly quantified. Given the limited z-resolution and temporal sampling (single plane imaging at 350 ms intervals), this conversion should be interpreted cautiously as a rough estimate rather than a robust cellular rate.

5. The model places Dynamin-2 and Endophilin B1 function at phagophore closure. However, Fig. 1I shows Dnm2-EndoB1 PLA signals mainly colocalize with early markers (Atg2, FIP200) and not with late/closure markers (STX17, Rab7). This suggests earlier recruitment than depicted, and the timing in Fig. 5H is therefore not fully supported by the presented data. Time-resolved or stage-specific experiments are needed to clarify the exact step of action.

6. Dynamin-2 has been proposed to function upstream in generating Atg9A vesicles from recycling endosomes (Takahashi et al., 2016; Soreng et al., 2018), in contrast to the current model in which Dnm2 acts at phagophore closure to retrieve Atg9A. The authors suggest different phagophore origins as a reconciliation, but this is not directly tested. Discriminating between ER-derived (DFCP1-positive) and recycling endosome-derived (Rab11-positive) sites of Atg9A accumulation in Dnm2 KO cells would help resolve these competing models. Triple-channel imaging of Atg9A, LC3, and either DFCP1 or Rab11 in WT and KO cells would clarify whether Atg9A accumulation is biased toward a specific membrane source.

Undefined abbreviations

© 2026. Published by The Company of Biologists under the terms of the Creative Commons Attribution License (https://creativecommons.org/licenses/by/4.0/).

* CCCP is used throughout the manuscript without ever being defined at first use in the results section.

* MEF - mouse embryonic fibroblast is used from the Results onward without definition at first use

* EBSS - Earle's balanced salt solution is used in the Results ("starved for four hours in EBSS") without ever being defined

* DKO - double knockout appears in without being defined in the main text at first use, only implied by context.

Author Contributions

The author contributions state "CIM, SI, JS and BR conducted experiments" but there is no author with initials BR in the author list. This discrepancy requires correction or clarification in the Author Contributions section.

Reviewer's Responses to Questions

Experimental quality
Are experiments performed using appropriate methods that will answer the question (or test the hypothesis or support the observations) posed by the authors? Is the right tool used for the job?
If 'No', please indicate reasons in Comments for Author box below.
Reviewer #1:

- Yes

Reviewer #2:

- Yes

Were the data analyzed using appropriate statistical tests?
If 'No', please indicate reasons in Comments for Author box below.
Reviewer #1:

- Yes

Reviewer #2:

- Yes

Reproducibility
Were experiments performed using adequate number of biological replicates?
If 'No', please indicate reasons in Comments for Author box below.
Reviewer #1:

- Yes

Reviewer #2:

- Yes

Is there sufficient raw data to assess the rigor of the analysis?
If 'No', please indicate reasons in Comments for Author box below.
Reviewer #1:

- Yes

Reviewer #2:

- Yes

© 2026. Published by The Company of Biologists under the terms of the Creative Commons Attribution License (https://creativecommons.org/licenses/by/4.0/).

Does the methods section provide sufficient detail to permit reproducibility?
If 'No', please indicate reasons in Comments for Author box below.
Reviewer #1:

- Yes

Reviewer #2:

- Yes

Completeness
Are the manuscript's conclusions supported by the data?
If 'No', please indicate reasons in Comments for Author box below.
Reviewer #1:

- Yes

Reviewer #2:

- No

Scholarship
Do the authors cite and discuss the merits of data that would argue for and against their conclusion?
If 'No', please indicate reasons in Comments for Author box below.
Reviewer #1:

- Yes

Reviewer #2:

- No

Does the manuscript title & abstract accurately reflect the contents of the manuscript, without hyperbole?
If 'No', please indicate reasons in Comments for Author box below.
Reviewer #1:

- Yes

Reviewer #2:

- Yes

First revision

Author response to reviewers' comments

We much appreciate the reviewers' comments and have made the suggested changes. This has strengthened the paper.

Reviewer 1: This manuscript presents an interesting and potentially important model in which Dnm2 promotes Atg9A retrieval from phagophores during autophagy. The study uses multiple complementary approaches and addresses a significant mechanistic question in the autophagy field, making it potentially suitable for publication. The overall conclusions are intriguing and potentially suitable for publication; however, three major issues should be addressed to strengthen the mechanistic conclusions, along with several minor revisions related to clarity, consistency, and figure presentation.

Major comments:

© 2026. Published by The Company of Biologists under the terms of the Creative Commons Attribution License (https://creativecommons.org/licenses/by/4.0/).

1. Missing genetic rescue is a major gap: A significant weakness of the study is the absence of genetic rescue experiments. The authors rely on knockout cells and pharmacological perturbations to support a role for Dnm2 in Atg9A retrieval, but do not demonstrate restoration of the phenotype by re-expression of Dnm2. This would be important to confirm the specificity of the phenotype. At a minimum, the central model would be significantly strengthened by the rescue of Atg9A retrieval defects with WT Dnm2.

We now include a rescue experiment, using Dyn2-eGFP (Addgene 34686) transfections of the Dnm2 KO) cells (new Fig. S3A). This experiment shows significantly reduced Atg9A-myc/HA-LC3 colocalization in cells expressing Dyn2-eGFP, indicating that off-target effects of the gene knockouts are unlikely.

2. The direct membrane scission mechanism remains inferred rather than demonstrated. While the data support a role for Dnm2 in Atg9A retrieval, the stronger claim that Dnm2 directly mediates membrane scission at phagophores remains somewhat inferential. The authors should either provide stronger mechanistic evidence supporting direct scission or temper the wording throughout the manuscript where Dnm2 is described as the scission factor.

The reviewer is correct to point out that a direct role in scission is primarily inferred from gain- and loss-of-function data. The most direct evidence we could provide is colocalization of Dnm2 spots with scission events in live-cell microscopy, but those experiments have technical limitations, as noted in another comment. Direct evidence would require an in vitro fission assay, for example, with GUVs, but it would be difficult to recreate conditions that faithfully reflect scission at an autophagosome. Given the range of approaches we used to test a role in scission, we still believe it is justified to propose this function, but we have tempered its phrasing in the manuscript.

3. The pulse-chase approach is elegant and potentially informative, but several controls are needed to support the interpretation that delayed Atg9A-p62 dissociation reflects defective Atg9A retrieval specifically.

* Verification that Vps34 inhibition effectively blocks autophagic flux.

* Demonstration that LC3 lipidation recovers after Vps34-IN1 washout

* Evidence that delayed Atg9A-p62 dissociation in Dnm2 KO cells does not simply reflect delayed recovery of autophagy, rather than a specific retrieval defect.

We now provide a blot showing that LC3-II levels are low in cells treated with VPS34inh but recover quickly and to similar extents in wildtype and mutant cells after washing out the inhibitor, thus demonstrating the blockage is caused by VPS34inh and that the differences in Atg9A-p62 dissociation are not due to delayed recovery (new Fig. S5C).

Minor comments:

1. Figure 1:
A. In the PLA experiment (1E, F), although the text states all autophagy-inducing conditions significantly increased PLA, statistics appear to be shown only for AntA. Please provide a statistical analysis for all conditions discussed.

We reviewed the statistics used throughout the paper and concluded that some experiments, including this one, should be analyzed using one-way ANOVA followed by Dunnett's post hoc test because they compare multiple treatments against a single control rather than against each other. This was done for Fig. 1 B,D, F, G and I, Fig. 5B and Fig. S1 B and D. The only change in significance was for Fig. 1F, where CCCP and Antimycin A both induce significant changes in the numbers of PLA spots/cell (** for each), while the increases for Rapamycin and starvation remained below the threshold for significance for the given number of replicates.

© 2026. Published by The Company of Biologists under the terms of the Creative Commons Attribution License (https://creativecommons.org/licenses/by/4.0/).

B. Related to results in Fig 1: Page 4, Lines 7-10: The sentence beginning "The increase in PLA spots…" appears redundant and could be removed.

As suggested, this sentence was removed.

C. Figure1 F-H: The presentation and labeling of the PLA data should be clarified and standardized. In Fig. 1F and G, y-axis terminology is inconsistent ("spots/cell" vs "PLA spots/cell"), and the legend alternates between "dots" and "spots"; consistent nomenclature should be used throughout. Panel G also refers to "relative numbers," which should be explicitly defined. In addition, the substantially different y-axis ranges between panels (0-50 vs 0-10) should be justified or harmonized to facilitate comparison. Fig. 1H, treatment conditions should be labeled directly on the figure, representative images for the untreated control should be included, and there was a decrease in the fraction of PLA spots associated with LAMP1. Is this a significant decrease?

As suggested, the Y-axis labeling in panel F was changed to PLA spots/cell and dots were changed to spots in the figure legend for consistency. Panel G legend reference to relative numbers was an error. They are absolute numbers. Sorry for the mistake. The difference in range between panel F (0-50) and panels G and I (0-10) is attributable to differences in the sensitivity of the batches of PLA reagents available to us at the time those experiments were performed. Because biological replicates for each experiment were performed with a single batch, the numbers can be compared within a single panel. We added a mention of the treatment to Panel H. This panel served merely to illustrate the methods used in Panels G and I, and not necessarily to corroborate the numbers or significance. The reduced association between PLA spots and LAMP1 was not significant.

2. Figures 2/3:

A. Figure 2B: I recommend labeling TOM20 RED and Hsp60 green directly on images. This would improve readability and make the images easier to interpret without requiring the reader to repeatedly refer back to the legend.

Agreed, that is a good idea. We did this now.

B. Figure 2C: Please address the apparent discrepancy between microscopy (complete signal loss) and Western blot (strong remaining WT signal at 24h) and indicate whether degradation between 0h and 24h is significant.

This discrepancy likely resulted from an unfortunate (non-representative) selection of images for panel B and blots for panel C, as also suggested by panel D, which shows substantial variability in the effects observed across experiments at 24 h. We have now replaced panel B with a more representative set of images. This does not affect the conclusion that mitophagy is not slower in Dnm2 KO cells. To the contrary, it may even be somewhat accelerated.

C. Use consistent labeling ("0 h" vs "untreated") across panels. (e.g., Fig. 2C vs 2D)

Thank you for noticing this. We relabeled the panels accordingly.

D. Figure 2E: WT and Dnm2 KO show additional bands on the tubulin blot that are not present in the EndoB KO or the DKO. Why is there an extra band?

There were 2 blots in this experiment. WT and Dnm2 KO extracts were on one blot and EndoB and DKO were on the other blot. The first one was incubated with a fresher antibody or washed for a shorter time, because the bands were more intense and some cross-reacting bands appeared. The main band is the correct size for tubulin, and the intensities are consistent within a set, justifying its use as a loading control.

E. Please use consistent nomenclature for Bafilomycin A throughout the manuscript, Fig. S2A: "BafA", other figures "Baf".

Thank you for noticing this. We relabeled the panels accordingly.

© 2026. Published by The Company of Biologists under the terms of the Creative Commons Attribution License (https://creativecommons.org/licenses/by/4.0/).

F. Page 5, line 8: Replace mutations in Dnm2 with loss of Dnm2 or Dnm2 knockout.

Thank you for this comment. The wording was corrected.

G. Page 5, lines 11-15: The interpretation that Fig. 3B demonstrates "more red spots" may overstate what is shown; panel B appears to support increased autophagosomes rather than specifically autolysosomes.

The reviewer correctly pointed out that 3B shows numbers of autophagosomes, while 3C shows autophagolysosomes. This was corrected in the text.

3. Figure presentation/consistency:

A. Figure Presentation: Throughout the manuscript, fluorophore identities should be indicated directly on the figures rather than only in the legends. In several panels (for example, Fig. 4A), the reader must infer which signal corresponds to each marker. I recommend labeling fluorophores within the figures using color-coded text matching the channels (e.g. Atg9A in green, LC3B in red), which would substantially improve readability and allow readers to interpret images without repeatedly referring back to the legends. Figure 4D provides a good example of this approach and could serve as a model for the rest of the manuscript.

As suggested, the identities of the fluorophores were added to each panel.

B. Supplement Fig. S3: Standardize "untreated" vs "DMSO" labeling and check for similar inconsistencies throughout.

Thank you for noticing this. We relabeled the panels accordingly.

C. Figure 4C: The blot is cropped from the right. Please provide a full, uncropped blot with complete protein labels.

We now include the right-hand cropped portion (fraction #22) and made sure the labeling is correct.

D. Please provide representative images for the Dynole 34-2 and Ryngo 1-23 experiments, potentially as supplemental data.

As requested, we now include representative images for the Dynole 34-2 and Ryngo 1-23 experiments in Fig. S5A.

E. Figure 5C would benefit from representative images accompanying quantification.

As requested, we now include representative images from the washout experiment in Fig. S5B.

4. In the Discussion, the authors state that five lines of evidence support the model that Dnm2 is essential for Atg9A retrieval. Please cite the corresponding figures for each line of evidence explicitly.

That is a good idea. We did that now.

5. Materials and methods: Rabbit anti-dynamin 1 is listed in the method section, although it was not used in this study. I also recommend including the catalog numbers of each antibody used for reproducibility.

Rabbit anti-dynamin 1 was removed from the methods. This antibody was used in an early version of the manuscript, in which we also showed that dynamins did not affect mitochondrial fission. This negative result was dropped since others have also shown the same lack of effect. We added a new Atg9A antibody from ProteinTech for the most recent experiment. A list is provided at the end of the Methods section.

© 2026. Published by The Company of Biologists under the terms of the Creative Commons Attribution License (https://creativecommons.org/licenses/by/4.0/).

Reviewer 2: General comments

This manuscript proposes that Dynamin-2 (Dnm2) and Endophilin-B1 (EndoB1) mediate retrieval of Atg9A from phagophores via membrane fission. The biological question is well-motivated. Atg9A is known to be absent from mature autophagosomes (Orsi et al., 2012) but the retrieval mechanism is unknown. The study's multi-method approach (PLA, live-cell imaging, subcellular fractionation, and pharmacological modulation) is a clear strength, and the convergence of these independent datasets is compelling. However, several key issues remain that need to be addressed.

Specific comments

1. Figure 5F-G shows that Atg9A levels decrease in Dnm2 KO cells during CCCP-induced mitophagy and are restored by Bafilomycin A1, indicating that Atg9A undergoes lysosomal degradation in the absence of Dnm2. However, Figures 2-3 suggest that mitophagy and bulk autophagic flux are maintained or even enhanced under these conditions. Given that Atg9A is implicated in supplying membrane for autophagosome formation, sustained depletion would be expected to limit autophagosome biogenesis over time. The manuscript does not address how autophagic activity is maintained despite this apparent loss. Possible explanations include compensatory upregulation of Atg9A synthesis, increased efficiency of Atg9A recycling, or the use of alternative membrane sources. Clarifying whether Atg9A levels recover over time or whether autophagy remains sustained during prolonged stress would help resolve this issue.

Under basal conditions, mutant and wild-type cells have similar Atg9A protein levels, suggesting that any additional Atg9A loss through basal autophagy in the mutant is small. Pilot experiments using cycloheximide to monitor Atg9A turnover and wortmannin to inhibit basal autophagy likewise showed no substantial difference between mutant and wild-type cells. Unfortunately, the rabbit polyclonal antibody used in our original experiments is no longer available, and a newer Proteintech monoclonal antibody did not produce sufficiently clean results to include in our manuscript.

We can, nevertheless, also consider what is already known about turnover rates. Atg9A has a half-life of 23.8 h in HeLa cells (approximately 2.9% turnover per hour) (Cambridge et al. 2011, J Proteome Res 10, 5275-84), although comparable data are unavailable for MEFs. Recent studies indicate that basal autophagy utilizes only a very small fraction of the large, cycling Atg9A pool in TGN, endosomal, and recycling membranes, potentially as little as one Atg9A vesicle per productive phagophore (Broadbent et al. 2023 J Cell Biol 222 e202210078 and Olivas et al. 2023 J Cell Biol 222 e202208088). Thus, even if Atg9A is mistargeted and degraded in Dnm2 KO cells, the resulting additional turnover is likely to be small under basal conditions and to constitute a negligible fraction of total turnover. Consistent with this interpretation, differences in Atg9A levels become apparent only when autophagic demand is substantially increased, such as during 8 h of CCCP-induced mitophagy. We therefore expect differences in Atg9A synthesis rates between mutant and wild-type cells to be very small under basal conditions. These considerations were added to the manuscript.

2. The authors note that anti-Atg9A immunofluorescence shows lower specificity in mouse cells compared to human cells. However, key colocalization analyses in Figures 4A-B rely on this antibody in MEFs without inclusion of an Atg9A knockout negative control. As a result, the specificity of the staining and therefore the interpretation of Mander's coefficients cannot be fully assessed. Inclusion of Atg9A KO MEFs as a negative control would be important to validate the signal and support the colocalization analysis.'

This was a misunderstanding due to a poor description on our part in the text. Fig. 4A-B shows IF with Atg9-myc and HA-LC3 constructs, while Fig. S3A-C shows live imaging with RFP-Atg9 and GFP-LC3. This is now clarified in the text and in the figure legend. The pulse chase experiments in Fig. 5C were conducted with IF for endogenous Atg9 in HeLa cells, because the Atg9 antibody did not work well in MEFs, as also explained in the text. Representative images are now included.

3. The key fractionation experiment (Fig. 4C) relies on Bafilomycin pretreatment to accumulate autophagosomes. However, Bafilomycin elevates lysosomal pH and induces organelle swelling, which can alter buoyant density independently of changes in protein composition. Therefore, the

© 2026. Published by The Company of Biologists under the terms of the Creative Commons Attribution License (https://creativecommons.org/licenses/by/4.0/).

enrichment of Atg9A, LC3-II, and p62 in intermediate fraction #14 in Dnm2 KO cells may reflect Bafilomycin-induced density shifts rather than altered autophagosomal content. The inclusion of a WT + Bafilomycin control (without Rapamycin) would help distinguish among these possibilities.

Thank you for the suggestion that Bafilomycin may have induced a density shift. This intriguing explanation is now included in the results section and raises a point for further discussion. The presence of Rab7 in this fraction suggests that it contains immature autophagolysosome intermediates. WT cells also have a small amount of LC3 and Rab7 in this fraction, but no Atg9. The increased levels of LC3, Rab7, and especially Atg9 in fraction 14 suggest that Dnm2 KO delays the maturation of these autophagolysosome intermediates into mature lysosomes. Critically, however, Atg9 is not normally present in autophagolysosomes (Judith et al. 2019 J Cell Biol 218, 1634-1652; Olivas et al. 2023 J Cell Biol 222; Orsi et al. 2012 Mol Biol Cell 23, 1860-73). Further investigation of Dnm2 function at this later stage is beyond the scope of the current paper.

4. The central mechanistic evidence (Fig. 5A) is based on overexpressed Dnm2-RFP, GFP-Atg9A, and BFP-LC3. Given that Dynamin-2 overexpression can alter membrane morphology (including tubulation), it is difficult to exclude the possibility that the observed events reflect overexpression artefacts rather than physiological behavior. The extrapolation from low-frequency events (1 in 3 videos in a single focal plane) to ~25 events per cell per minute relies on assumptions about spatial and temporal sampling that are not explicitly quantified. Given the limited z-resolution and temporal sampling (single plane imaging at 350 ms intervals), this conversion should be interpreted cautiously as a rough estimate rather than a robust cellular rate.

The reviewer is correct to point out the system's limitations. We added the phrase "rough estimate" to the sentence describing these observations to make these limitations clearer.

5. The model places Dynamin-2 and Endophilin B1 function at phagophore closure. However, Fig. 1I shows Dnm2-EndoB1 PLA signals mainly colocalize with early markers (Atg2, FIP200) and not with late/closure markers (STX17, Rab7). This suggests earlier recruitment than depicted, and the timing in Fig. 5H is therefore not fully supported by the presented data. Time-resolved or stage-specific experiments are needed to clarify the exact step of action.

The reviewer was correct to point out that the colocalization of Dnm2-EndoB1 PLA spots with FIP200 and Atg2 (Fig. 1I) suggests an earlier association between Dnm2 and EndoB1 with the growing phagophore than depicted in our model (Fig. 5H). We have now updated the drawing to include this earlier association.

6. Dynamin-2 has been proposed to function upstream in generating Atg9A vesicles from recycling endosomes (Takahashi et al., 2016; Soreng et al., 2018), in contrast to the current model in which Dnm2 acts at phagophore closure to retrieve Atg9A. The authors suggest different phagophore origins as a reconciliation, but this is not directly tested. Discriminating between ER-derived (DFCP1-positive) and recycling endosome-derived (Rab11-positive) sites of Atg9A accumulation in Dnm2 KO cells would help resolve these competing models. Triple-channel imaging of Atg9A, LC3, and either DFCP1 or Rab11 in WT and KO cells would clarify whether Atg9A accumulation is biased toward a specific membrane source.

The reviewer correctly notes that we did not address the origin of Atg9a vesicles or the sites of autophagosome assembly, both of which are subjects of competing models. The Wang and Simonsen labs (Takahashi et al., 2016; Soreng et al., 2018) suggest that Atg9a vesicles originate from early endosomes, whereas the Tooze lab proposes that they could also originate from the Golgi (Judith et al., 2019). We found that Dnm2/EndoB PLA spots do not colocalize with Rab11, suggesting that this pair of proteins is not responsible for generating Atg9a vesicles at early endosomes. However, Dnm2 and EndoB KO cells, as well as DKO cells, remain capable of autophagy, indicating that these proteins are not required for this process. We did not investigate these discrepancies because the reported differences in the origins of Atg9a vesicles and the sites of autophagosome assembly do not alter the roles of Dnm2 and EndoB in autophagosome dynamics described in our manuscript.

Undefined abbreviations

© 2026. Published by The Company of Biologists under the terms of the Creative Commons Attribution License (https://creativecommons.org/licenses/by/4.0/).

* CCCP is used throughout the manuscript without ever being defined at first use in the results section.

* MEF - mouse embryonic fibroblast is used from the Results onward without definition at first use

* EBSS - Earle's balanced salt solution is used in the Results ("starved for four hours in EBSS") without ever being defined

* DKO - double knockout appears in without being defined in the main text at first use, only implied by context.

Author Contributions

The author contributions state "CIM, SI, JS and BR conducted experiments" but there is no author with initials BR in the author list. This discrepancy requires correction or clarification in the Author Contributions section.

Thank you for pointing out these errors. They were duly corrected.

Second decision letter

MS ID#: bio.062632R1

MS Title: Dynamin-2 promotes Atg9A retrieval from phagophores during autophagy

Authors: Alex Van der Bliek; Andrew Caliri; Alejandro Martorell Riera; Akash Saha; Panagiota Kolitsida; Cinta Iriondo Martinez; Samuel Itskanov; Janos Steffen; Carla M. Koehler

I am happy to tell you that your manuscript has been accepted for publication in Biology Open, pending our standard publication integrity checks. It was accepted on 27th August 2026.

© 2026. Published by The Company of Biologists under the terms of the Creative Commons Attribution License (https://creativecommons.org/licenses/by/4.0/).
